# P2X7 receptor inhibition prevents atrial fibrillation in rodent models of depression

**DOI:** 10.1093/europace/euae022

**Published:** 2024-01-23

**Authors:** Tianxin Ye, Yunping Zhou, Jinxiu Yang, Fangcong Yu, Zhuonan Song, Jiaran Shi, Longbo Wang, Zhouqing Huang, Bo Yang, Xingxiang Wang

**Affiliations:** Department of Cardiology, The First Affiliated Hospital, Zhejiang University School of Medicine, #79 Qingchun Road, Hangzhou, Zhejiang Province 310003, PR China; Department of Cardiology, The First Affiliated Hospital, Zhejiang University School of Medicine, #79 Qingchun Road, Hangzhou, Zhejiang Province 310003, PR China; Department of Cardiology, The First Affiliated Hospital, Zhejiang University School of Medicine, #79 Qingchun Road, Hangzhou, Zhejiang Province 310003, PR China; Department of Cardiology, The First Affiliated Hospital, Zhejiang University School of Medicine, #79 Qingchun Road, Hangzhou, Zhejiang Province 310003, PR China; Department of Cardiology, The First Affiliated Hospital, Zhejiang University School of Medicine, #79 Qingchun Road, Hangzhou, Zhejiang Province 310003, PR China; Department of Cardiology, The First Affiliated Hospital, Zhejiang University School of Medicine, #79 Qingchun Road, Hangzhou, Zhejiang Province 310003, PR China; Department of Cardiology, The First Affiliated Hospital, Zhejiang University School of Medicine, #79 Qingchun Road, Hangzhou, Zhejiang Province 310003, PR China; Department of Cardiology, The First Affiliated Hospital of Wenzhou Medical University, 2 Fuxue Road, Wenzhou, Zhejiang Province 325000, PR China; Department of Cardiology, Renmin Hospital of Wuhan University, 238 Jiefang Road, Wuchang District, Wuhan 430060, PR China; Cardiovascular Research Institute, Wuhan University, 238 Jiefang Road, Wuchang District, Wuhan 430060, PR China; Hubei Key Laboratory of Cardiology, Wuhan 430060, PR China; Department of Cardiology, The First Affiliated Hospital, Zhejiang University School of Medicine, #79 Qingchun Road, Hangzhou, Zhejiang Province 310003, PR China

**Keywords:** P2X7 receptor, Depression, Atrial fibrillation, Inflammation

## Abstract

**Aims:**

Depression, the most prevalent psychiatric disorder, is associated with the occurrence and development of atrial fibrillation (AF). P2X7 receptor (P2X7R) activation participates in the development of depression, but little attention has been given to its role in AF. This study was to investigate the effects of P2X7R on AF in depression models.

**Methods and results:**

Lipopolysaccharide (LPS) and chronic unpredictable stress (CUS) were carried out to induce depression in rodents. Behavioural assessments, atrial electrophysiological parameters, electrocardiogram (ECG) parameters, western blot, and histology were performed. Atrial fibrillation inducibility was increased in both LPS- and CUS-induced depression, along with the up-regulation of P2X7R in atria. CUS facilitated atrial fibrosis. CUS reduced heart rate variability (HRV) and increased the expression of TH and GAP43, representing autonomic dysfunction. Down-regulation of Nav1.5, Cav1.2, Kv1.5, Kv4.3, Cx40, and Cx43 in CUS indicated the abnormalities in ion channels. In addition, the expression levels of TLR4, P65, P-P65, NLRP3, ASC, caspase-1, and IL-1β were elevated in depression models. Pharmacological inhibitor (Brilliant Blue G, BBG) or genetic deficiency of P2X7R significantly mitigated depressive-like behaviours; ameliorated electrophysiological deterioration and autonomic dysfunction; improved ion channel expression and atrial fibrosis; and prevented atrial NLRP3 inflammasome activation in the pathophysiological process of AF in depression models.

**Conclusion:**

LPS or CUS induces AF and promotes P2X7R-dependent activation of NLRP3 inflammasome, whereas pharmacological P2X7R inhibition or P2X7R genetic deficiency prevents atrial remodelling without interrupting normal atrial physiological functions. Our results point to P2X7R as an important factor in the pathology of AF in depression.

## Graphical Abstract

**Figure:**
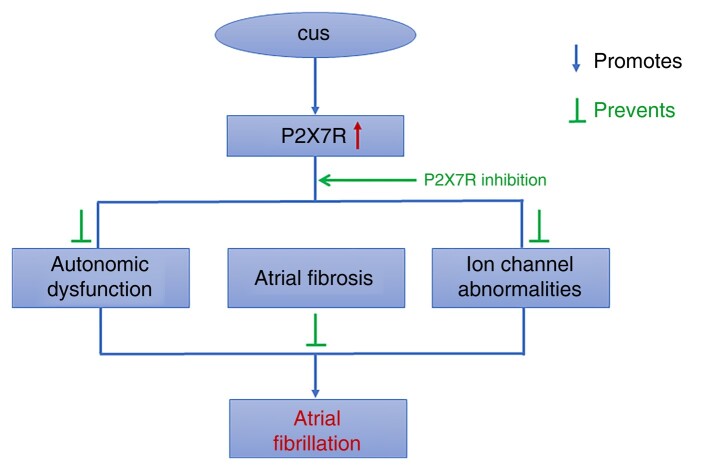

**What’s new?**

- P2X7 receptor (P2X7R) was up-regulated in atria in depression models.
- P2X7R inhibition mitigated depressive-like behaviours and prevented atrial fibrillation (AF) in depression models.
- P2X7R inhibition mitigated autonomic dysfunction, atrial fibrosis, and ion channel expression in depression models.
- Targeting P2X7R could be a promising target for AF in depression.

## Introduction

Depression, also known as major depressive disorder, is the most prevalent psychiatric mood disorder affecting over 300 million individuals worldwide, which may last a long time and lead to devastating personal, social, and economic burdens globally.^1^ In the past two decades, apart from the effects of depression on mental conditions, emerging studies have revealed the bidirectional association between depression and cardiovascular diseases (CVD).^2^ Patients with CVD such as myocardial infarction (MI), heart failure (HF), and stroke had a higher prevalence of depressive symptoms.^3,4^ Baseline depressive symptoms were closely related to the incidence of CVD;^5^ in addition, patients with depression had a strong and positive genetic correlation with CVD such as HF.^6^ Previous work has also indicated the potential associations between depression and arrhythmias including atrial fibrillation (AF),^7^ which may in part explain the positive correlation between depression and the increased risk of cardiac-related or all-cause death.^2^

AF, the most common cardiac arrhythmia affecting about 2–4% of adults, can cause considerable deterioration in quality of life and promote major cardiac events such as ischaemic stroke, HF, and even all-cause mortality, leading to an enormous burden to healthcare systems globally.^8^ Psychological distress such as depression occurs in patients with AF; enhances symptom severity of AF; complexes management; and even increases the risk of adverse cardiovascular outcomes.^9,10^ The presence of depression increased the incidence of all-cause mortality in patients with AF and was associated with AF recurrence after ablation.^7,11^ More importantly, pre-existing depression was observed to increase the risk of new-onset AF.^12^ Our previous research has also pointed out that depression facilitates the initiation of ventricular arrhythmia (VA) in a rat model of chronic unpredictable stress (CUS).^13^ These studies provide evidence for the association between depression and arrhythmia; however, to our knowledge, less attention has been given to AF in depression and its potential mechanisms despite that depression may cause chronic inflammation and cardiac autonomic dysfunction, which also participate in the occurrence and maintenance of AF.^2,13,14^

P2X7 receptor (P2X7R), one of the P2X ion channel receptors, is widely expressed in almost all organs and mediates mainly inflammation as well as cellar death. High extracellular concentrations of adenosine triphosphate (ATP) can activate P2X7R, eliciting Ca^2+^ and Na^+^ influx, as well as K^+^ efflux. Previous pre-clinical studies have revealed that P2X7R is involved in the process of depression.^15^ P2X7R antagonists such as A-804598 and Brilliant Blue G (BBG) mitigated depressive behaviours in the CUS-induced rodent model of depression.^16,17^ Emerging studies have also identified that P2X7R participates in the development of CVD, and strategies targeting P2X7R are beneficial in CVD in pre-clinical models such as atherosclerosis, MI, and fibrosis, with regulation of inflammation identified as potential mechanisms.^18^ Nod-like receptor family pyrin domain containing 3 (NLRP3) inflammasome has been identified to be associated with the initiation of AF;^19,20^ however, as an important upstream effector of NLRP3 inflammasome, little attention has been given to the role of P2X7R in AF. Therefore, in this study, we mainly investigated the effects of P2X7R on AF in rodent models of depression and explored the underlying mechanisms.

## Methods

Detailed descriptions of methods were shown in the Supplementary material.

### Animals

Male Sprague-Dawley (SD) rats (180–220 g), wild-type (WT) C57BL/6 (8–12 weeks) and P2X7R-knockout (KO) mice were housed under standard conditions (12 h light/dark cycle), allowing free access to food and water (unless indicated if special environment was needed). Animal studies were approved by the Animal Ethics Committee of the Tongren Hospital of Wuhan University (Wuhan Third Hospital; approval number: SY2020-042) and were conducted conformed to the NIH Guide for the Care and Use of Laboratory Animals.

### Models of depression

#### LPS-induced depression

Lipopolysaccharide (LPS), a potent activator of the immune system, is widely used for inflammation-associated depression in rodents.^21^ Rats were randomly grouped as follows: (i) CTL group: saline; (ii) LPS group: lipopolysaccharide (LPS); (iii) LPS + B30 group: LPS + 30 mg/kg BBG; and (iv) LPS + B50 group: LPS + BBG (50 mg/kg). LPS (*Escherichia coli* 055:B5, sigma) was solubilized in saline and injected (0.5 mg/kg) once daily for a total of 7 days. BBG (sigma) was solubilized in saline and administered at a dose of 30 or 50 mg/kg daily for 7 days. LPS and BBG were injected intraperitoneally (ip).

#### Chronic unpredictable stress-induced depression

CUS is the most effective and commonly used rodent model of depression in reflecting disease complexity.^22^ In brief, rats or mice were exposed to one of the following stressors for 28 consecutive days: (1) cage tilted at 45°C for 24 h, (2) moist bedding for 24 h, (3) behavioural restriction for 2 h, (4) ice water swimming at 4°C for 5 min, (5) hot water swimming at 42°C for 5 min, (6) fasting for 24 h, (7) water deprivation for 24 h, (8) tail pinched for 1 min (1 cm from the tail root), (9) cage shaken for 15 min, (10) overnight illumination, (11) predator sounds for 30 min, and (12) noise for 3 h. Supplementary material online, *Table S1* showed the details of the stressors and duration of the CUS paradigm. The stressors were performed at random times during the day with the same stimulus not occurring consecutively to maximize the unpredictability of the stressor.

In experiments of CUS-induced rats, animals were randomly divided into four groups as follows: (i) CTL group: saline; (ii) CTL + B group: BBG (30 mg/kg); (iii) CUS group: 4-week CUS + saline; and (iv) CUS + B group: 4-week CUS + BBG (30 mg/kg). For CTL + B and CUS + B rats, BBG was given once (ip) daily for 28 days starting on Day 1 of CUS construction. The CTL and CUS rats were given equal amounts of saline.

In experiments of CUS-induced mice, WT C57BL/6 and P2X7R-KO mice were randomly divided as follows: (i) CTL-WT group: WT mice; (ii) CTL-KO group: P2X7R-KO mice; (iii) CUS-WT group: 4-week CUS in WT mice; and (iv) CUS-KO group: 4-week CUS in P2X7R-KO mice. Control animals received no stressors.

The 1-week CUS induction was also carried out in rats to establish a cause-effect relation between depression and AF.

### Behavioural tests

#### Sucrose preference test

The sucrose preference test (SPT) was conducted to evaluate stress-induced anhedonia.

#### Forced swimming test

The forced swimming test (FST) was performed to measure the immobility to assess behaviour despair.

### Body weight

Animal body weight was measured after 4-week CUS in rats.

### Surface electrocardiogram

Rats were anaesthetized and electrocardiogram (ECG; lead II) was recorded using the PowerLab system (4/35, AD Instruments, Australia).

#### Electrocardiogram parameters

The average P-wave duration, the shortest P-wave duration (Pmin), the longest P-wave duration (Pmax), P-wave dispersion, and P–R interval were calculated manually via LabChart 8.0 software. P-wave dispersion was obtained by subtracting the Pmin from the Pmax.

#### Heart rate variability

Heart rate variability (HRV) was measured in both time and frequency domain indicators. Time domain indicators included heart rate (HR), mean RR interval, the standard deviation of normal RR intervals (SDNN), and the square root of the mean squared differences of successive RR intervals (RMSSD). Low frequency (LF), high frequency (HF), and LF/HF ratio belonged to frequency domain indicators. HF and LF bands were shown in Supplementary material online, *Table S2*.

### Atrial electrophysiological measurement

Animals were anaesthetized and heparinized. Hearts were perfused using the Langerdorff technique (AD Instruments, Dunedin, New Zealand). Tissue ECG and epicardial monophasic action potential (MAP) from the left atrial appendage (LAA) were recorded after a stabilization period of 10 min. The S1–S2 programme consisted of eight consecutive basic stimuli S1 [pacing cycle length (PCL): 200 ms] and one preceding stimulus S2, and the PCL of S1–S2 was gradually shortened from 100 to 1 ms. Effective refractory period (ERP) was determined as the longest S1–S2 interval that failed to catch atrial activity. Atrial activation latency (AL) referred to the time required from the onset of each S2 to the maximum dV/dt during the action potential upstroke of each paced beat. The S1–S1 stimulation procedure (PCL: 200 ms) was conducted with 10 stimuli. The action potential duration (APD) was measured at 90% repolarization (APD_90_). Atrial arrhythmias were induced by burst pacing. A rapid irregular atrial rhythm with an irregular ventricular response lasting at least 2 s was considered AF, and a regular atrial tachyarrhythmia was determined as atrial flutter (AFL). The PowerLab system (AD Instruments) and LabChart 8.0 software were applied to record and analyse all the signals.

### Masson's trichrome staining

The left atrium was extracted and fixed with 4% paraformaldehyde and embedded in paraffin. Masson’s trichrome method was used to observe the distribution and deposition of myocardial collagen. ImageJ software was used to analyse the degree of fibrosis.

### Immunofluorescence

Primary antibodies include P2X7R (diluted 1:200, Santa Cruz), growth-associated protein 43 (GAP43, diluted 1:200; Affinity), and tyrosine hydroxylase (TH, diluted 1:200; Affinity). P2X7R antibody (diluted 1:200, Santa Cruz) and Cardiac troponin T antibody (diluted 1:100; Affinity) were employed for double immunofluorescent staining in atrial tissues.

### Western blot

The primary antibodies were listed in Supplementary material online, *Table S3*.

### Statistical analysis

**Figure 1:**
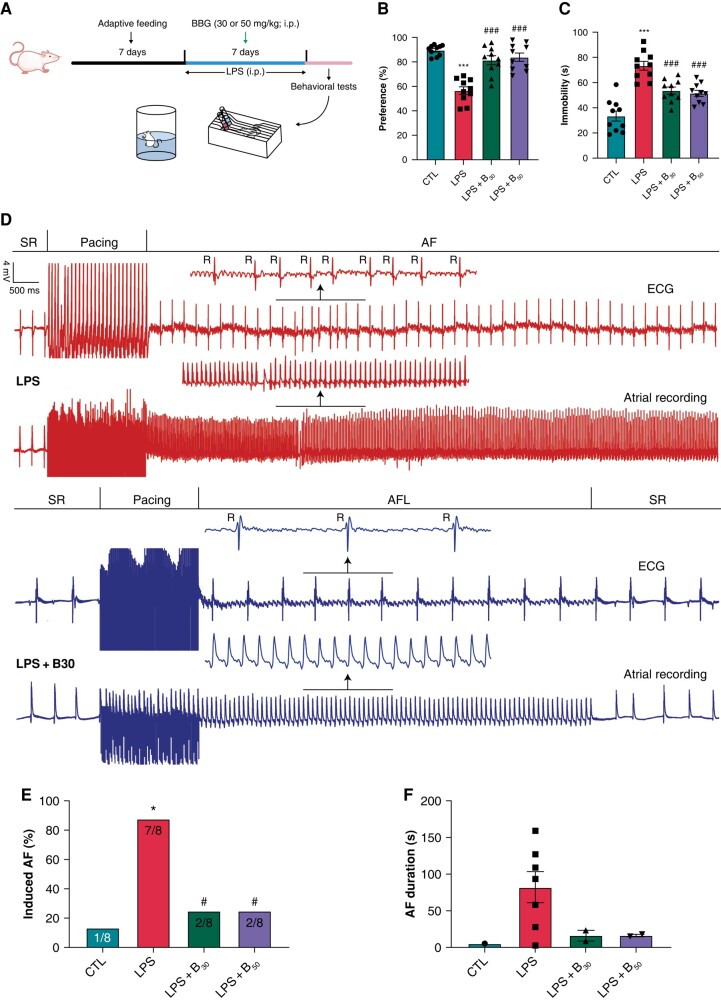
Depressive-like behaviours and AF inducibility in LPS-induced depression. (*A*) Schematic diagram of the experimental procedure. (*B*) Sucrose preference in the SPT. *n* = 10 per group. (*C*) Immobility in the FST. *n* = 10 per group. (*D*) Representative recordings of burst pacing protocol. (*E* and *F*) Inducibility and duration of AF, respectively. *n* = 8 per group. *P < 0.05, ***P < 0.001 vs. CTL; #*P* < 0.05, ###*P* < 0.001 vs. LPS. SPT, sucrose preference test; FST, forced swimming test; SR, sinus rhythm; R, R-wave; AFL, atrial flutter; AF, atrial fibrillation.

Continuous variables were expressed as mean ± SEM. The distribution of data was assessed using the Shapiro–Wilk test. The unpaired two-tailed Student’s *t*-test, Welch correction *t*-test, or Mann–Whitney *U* test was used for comparison between two groups when appropriate. Categorical variables were expressed as percentages. Atrial electrophysiological measurements were not performed in all subjects as the perfused hearts will not be used for other experiments such as histology or molecular measurements. Fisher’s exact test was used to analyse the AF inducibility in *Figures 1E* (*n* = 8 per group), *5G* (*n* = 10 per group), and *11G* (*n* = 8 per group), and Supplementary material online, *Figure S6C* (*n* = 6 per group). Differences among more than two groups were compared using one-way ANOVA followed by Bonferroni’s *post hoc* test or Kruskal–Wallis test as appropriate. The *P*-value of <0.05 was regarded as statistically significant.

## Results

### P2X7R inhibition improved depressive-like behaviours in LPS-induced rats

*Figure 1A* showed the paradigm of the LPS-induced depression model. Sucrose preference was significantly reduced in LPS rats (56.61 ± 2.91%) vs. controls (89.57 ± 1.28%) in the SPT. Immobility increased significantly from 33.51 ± 3.86 s (controls) to 73.47 ± 3.33 s in LPS rats in the FST. The reduced sucrose preference and increased immobility were prevented by BBG in comparison to LPS rats (all *P* < 0.001; *Figure 1B* and *C*).

### P2X7R inhibition ameliorated atrial fibrillation in LPS-induced rats

Later, we performed electrophysiological experiments to assay arrhythmogenesis in LPS-induced depression. The representative sample of the burst pacing protocol was displayed in *Figure 1D*. AF inducibility was significantly increased in LPS rats (seven of eight showing induced AF; 87.5%) than in controls (one of eight, 12.5%; *P* < 0.05), and AF duration tended to be longer in LPS rats compared to controls. BBG at two dosages prevented the alterations (*Figure 1E* and *F*).

### P2X7R inhibition prevented the up-regulation of P2X7R and the activation of NLRP3 inflammasome in LPS-induced rats

**Figure 2:**
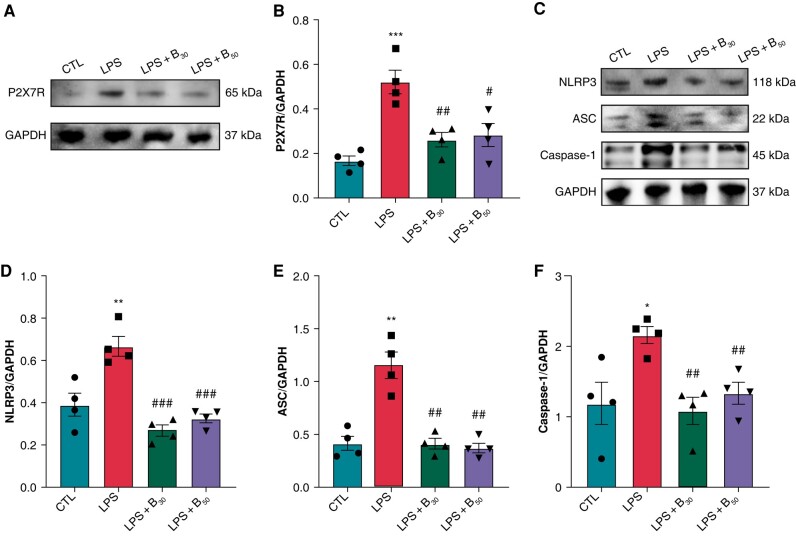
Expression of P2X7R and NLRP3 inflammasome-related indicators in LPS-induced depression. (*A* and *B*) Immunoblotting and expression ratio of P2X7R, respectively. *n* = 4 per group. (*C*–*F*) Immunoblotting and expression ratio of NLRP3, ASC, and Caspase-1, respectively. *n* = 4 per group. *P < 0.05, **P < 0.01, ***P < 0.001 vs. CTL; #*P* < 0.05, ##*P* < 0.01, ###*P* < 0.001 vs. LPS.

NLRP3 inflammasome, an important downstream effector of P2X7R, has been implicated in the pathogenesis of depression and AF. Therefore, the effect of P2X7R on NLRP3 inflammasome activation was investigated in atria under depression. As shown in *Figure 2A* and *B*, P2X7R was significantly elevated in the LPS group compared to controls. BBG significantly prevented the up-regulation of P2X7R both in the LPS + B30 and LPS + B50 group. Double immunofluorescent staining in atrial tissues with P2X7R and Cardiac troponin T revealed the increased P2X7R expression in cardiomyocytes of LPS rats, whereas BBG treatment could prevent the up-regulation of P2X7R (see Supplementary material online, *Figure S1*). Protein expression levels of NLRP3 inflammasome components including NLRP3, Caspase-1, and apoptosis-associated speck-like protein containing a CARD (ASC) were significantly increased in LPS-induced rats, whereas BBG significantly prevented these alterations in both LPS + B30 and LPS + B50 groups (*Figure 2C*–*F*).

### P2X7R inhibition improved depressive-like behaviours and prevented the up-regulation of atrial P2X7R in chronic unpredictable stress-induced rats

**Figure 3:**
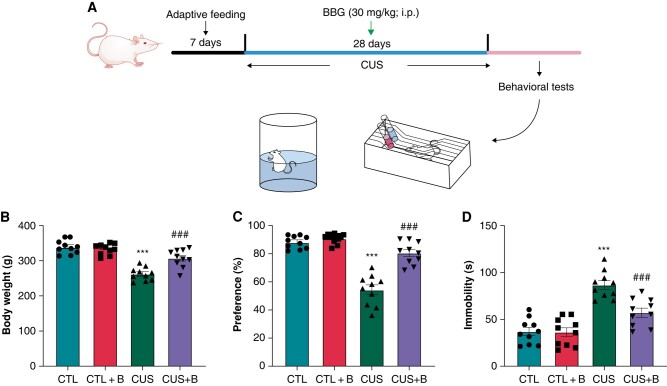
Depressive-like behaviours in CUS-induced rats. (*A*) Schematic diagram of the experimental procedure. (*B*) Body weight. *n* = 10 per group. (*C*) Sucrose preference in the SPT. *n* = 10 per group. (*D*) Immobility in the FST. *n* = 10 per group. ***P < 0.001 vs. CTL; ###*P* < 0.001 vs. CUS. SPT, sucrose preference test; FST, forced swimming test.

To further validate whether P2X7R is involved in AF in depression, the CUS-induced depression model was established. As BBG at the dose of 30 mg/kg has similar effects on behaviour tests and electrophysiological parameters to that of 50 mg/kg, we selected 30 mg/kg of BBG for the subsequent experiments. *Figure 3A* outlined the paradigm of CUS-induced depression in rats. CUS caused a significant reduction in body weight (262.5 ± 5.62 g) vs. controls (339.5 ± 5.82 g) (*Figure 3B*). Similar to results in LPS-induced depression, *Figure 3C* and *D* indicated that CUS led to a decline in sucrose preference (54.33 ± 3.25 vs. 88.27 ± 1.47% in controls, *P* < 0.001) in the SPT and an increase in immobility (86.52 ± 4.40 vs. 37.12 ± 4.06 s in controls, *P* < 0.001) in the FST, which revealed a successful establishment of depression model in CUS. BBG treatment significantly mitigated body weight and depressive-like behaviours (all *P* < 0.001, *Figure 3B*–*D*).

**Figure 4:**
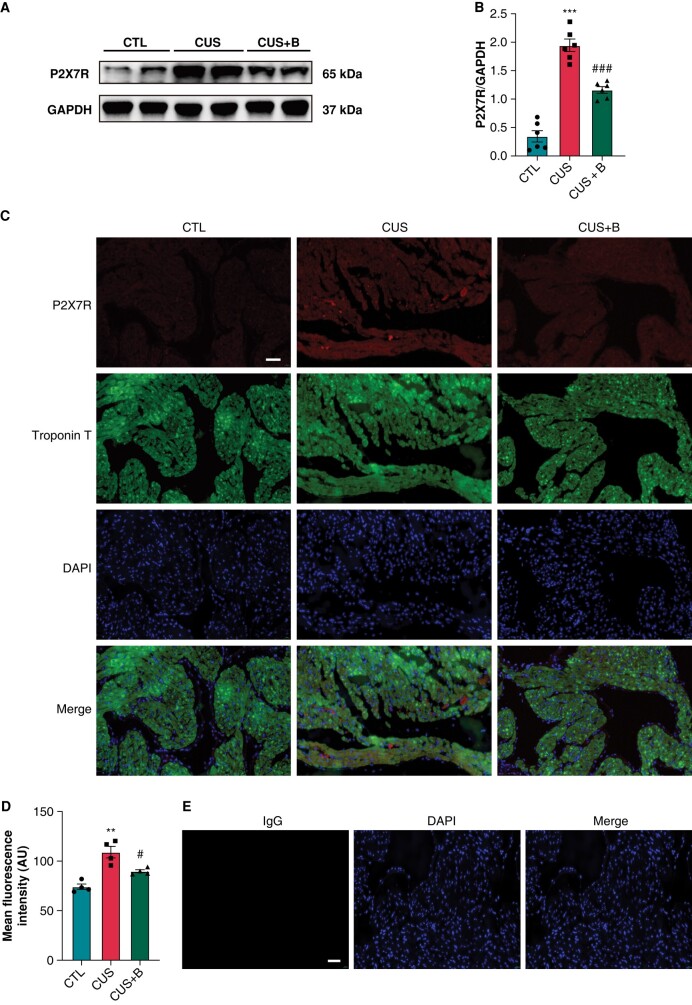
Expression of P2X7R in CUS-induced rats. (*A* and *B*) Immunoblotting and expression ratio of P2X7R, respectively. *n* = 6 per group. (*C* and *D*) Representative images of double-immunofluorescent labelling of P2X7R and Cardiac troponin T, and the mean fluorescence intensity of P2X7R, respectively. *n* = 4 per group. (*E*) Negative control was performed using specific IgG instead of the P2X7R antibody. Scale bar: 50 μm. **P < 0.01, ***P < 0.001 vs. CTL; #*P* < 0.05, ###*P* < 0.001 vs. CUS. AU, arbitrary units.

The protein expression of P2X7R was significantly up-regulated in CUS (1.95 ± 0.11) vs. controls (0.35 ± 0.10); BBG prevented the up-regulation of P2X7R compared to CUS rats (*P* < 0.001) (*Figure 4A* and *B*). Double immunofluorescent staining in atrial tissues with P2X7R and Cardiac troponin T was further performed to detect P2X7R expression in cardiomyocytes. We found the increased expression of P2X7R in cardiomyocytes in CUS compared to controls, which was prevented by BBG (*Figure 4C* and *D*).

### P2X7R inhibition prevented atrial arrhythmia in chronic unpredictable stress-induced rats

**Figure 5:**
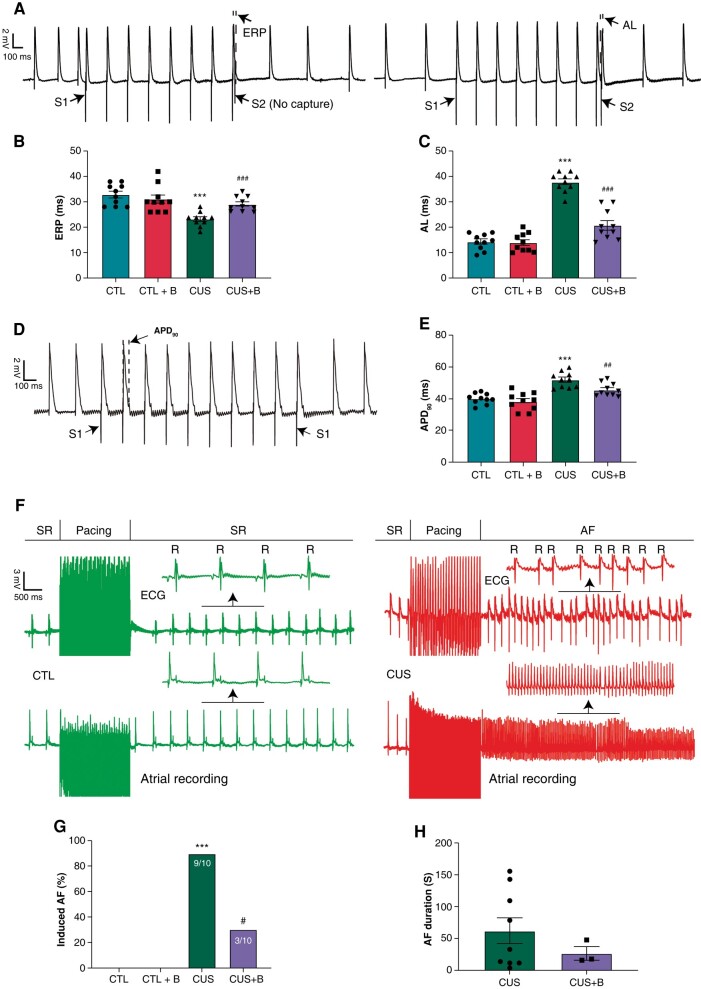
Electrophysiological phenotypes in CUS-induced rats. (*A*) Representative recordings of MPA in S1–S2 stimulation protocol. (*B* and *C*) ERP and AL, respectively. *n* = 10 per group. (*D*) Representative recording of MAP in S1–S1 stimumlation protocol. (*E*) APD_90_, *n* = 10 per group. (*F*) Representative recordings of burst pacing protocol. (*G*) Inducibility of AF. *n* = 10 per group. (*H*) Duration of AF. *n* = 9 in CUS and *n* = 3 in CUS + B. ***P < 0.001 vs. CTL; #*P* < 0.05, ##*P* < 0.01, ###*P* < 0.001 vs. CUS. ERP, effective refractory period; AL, atrial latency; MAP, monophasic action potential; APD_90_, action potential duration at 90% repolarization; SR, sinus rhythm; R, R-wave; AF, atrial fibrillation.

*Figure 5A* showed the representative recordings of the MAP after S1–S2 stimulation. CUS led to a shortening of ERP (23.20 ± 0.86 vs. 32.80 ± 1.24 ms in controls, *P* < 0.001) and a prolongation of AL (37.80 ± 1.15 vs. 14.30 ± 0.95 ms in controls, *P* < 0.001) (*Figure 5B* and *C*). *Figure 5D* was the representative recording of the MAP after S1–S1 stimulation. Atrial APD_90_ was significantly prolonged in CUS rats (51.98 ± 1.48 ms) vs. controls (40.22 ± 1.00 ms) (*Figure 5E*). BBG treatment significantly prevented these changes. *Figure 5F* showed the typical samples of burst pacing protocol. AF inducibility was substantially greater in CUS rats (9 of 10; 90%) than in CTL rats (0 of 10; 0%); AF inducibility was prevented by BBG co-administration in CUS (*Figure 5G*). Although the statistical difference was not significant, numerically AF duration was longer in CUS rats vs. CUS + B rats (*Figure 5H*). These data showed that CUS exhibited similar atrial electrophysiological characteristics to those of LPS-challenged rats. BBG prevented the detrimental effects of CUS on atrial electrophysiological phenotypes.

### P2X7R inhibition prevented the abnormalities in P-wave parameters in chronic unpredictable stress-induced rats

**Figure 6:**
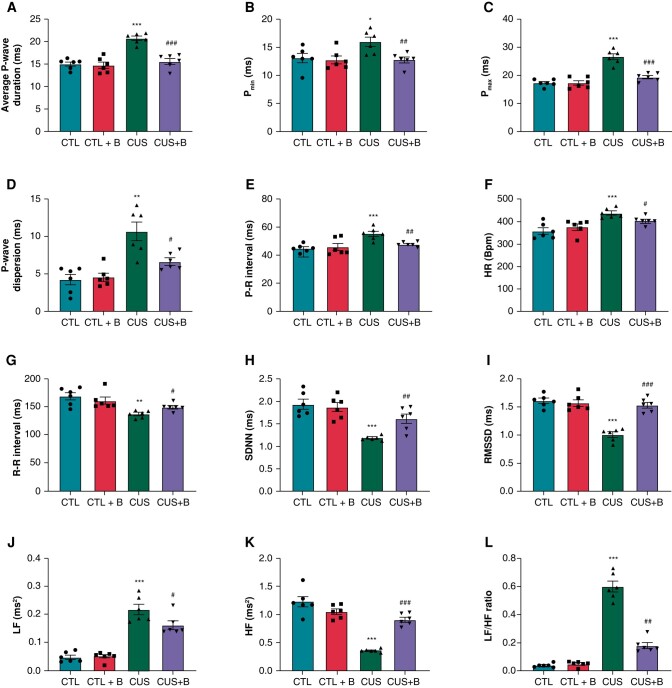
ECG and HRV parameters in CUS-induced rats. (*A*–*E*) Average *P*-average duration, Pmin, Pmax, P-wave dispersion, and P–R interval, respectively. *n* = 6 per group. (*F*–*L*) HR, R–R interval, SDNN, RMSSD, LF, HF, and LF/HF, respectively. *n* = 6 per group. *P < 0.05, **P < 0.01, ***P < 0.001 vs. CTL; #*P* < 0.05, ##*P* < 0.01, ###*P* < 0.001 vs. CUS. HRV, heart rate variability.

As shown in *Figure 6A*–*E*, CUS rats produced significant increases in average *P*-wave duration, Pmin, Pmax, P-wave dispersion, and P–R interval compared to the CTL group. BBG significantly prevented theses abnormal alterations in CUS + B rats. The representative ECG trace used for analysis was displayed in Supplementary material online, *Figure S2*.

### P2X7R inhibition alleviated cardiac autonomic dysfunction in chronic unpredictable stress-induced rats

**Figure 7:**
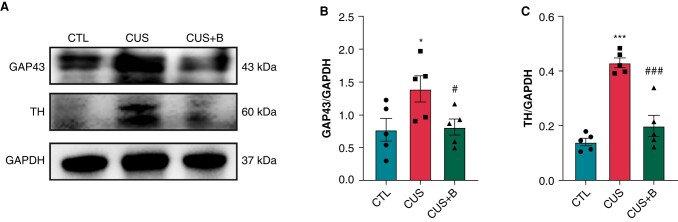
Expression of GAP43 and TH in CUS-induced rats. (*A*–*C*) Immunoblotting and expression ratio of GAP43 and TH, respectively. *n* = 5 per group. *P < 0.05, ***P < 0.001 vs. CTL; #*P* < 0.05, ###*P* < 0.001 vs. CUS.

HRV was measured to assess autonomic nervous system (ANS) function in CUS. A decrease in HRV usually implies parasympathetic inhibition and/or sympathetic enhancement. A visible shortening of RR interval, SDNN, HF, and RMSSD, as well as the increase in HR, LF, and LF/HF, were observed in CUS rats (*Figure 6F*–*L*). Moreover, the protein expression levels of GAP43 and TH were significantly increased in CUS rats (*Figure 7A*–*C*). Immunofluorescence also verified the elevated expression of GAP43 and TH in CUS (see Supplementary material online, *Figure S3*), suggesting autonomic dysfunction in the CUS-induced rat model of depression. Inhibition of P2X7R significantly attenuated the deterioration of HRV, and TH and GAP43 expression, indicating its role in regulating cardiac autonomic function.

### P2X7R inhibition mitigated atrial fibrosis in chronic unpredictable stress-induced rats

**Figure 8:**
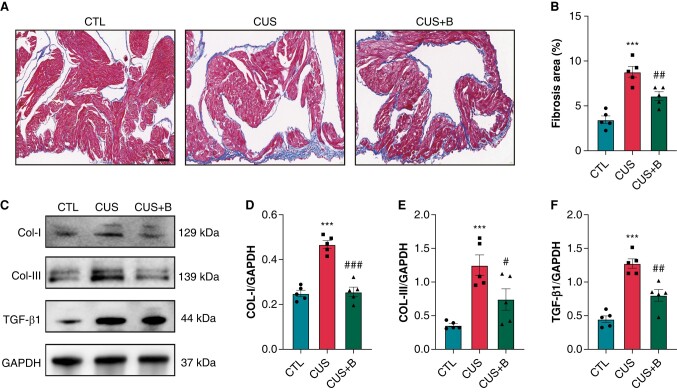
Atrial fibrosis in CUS-induced rats. (*A*–*B*) Representative images of Masson staining in atria and quantification of the fibrotic area, respectively. *n* = 5 per group. Scale bar: 100 μm. (*C*–*F*) Immunoblotting and expression ratio of COL-I, COL-III, and TGF-β, respectively. *n* = 5 per group. ***P < 0.001 vs. CTL; #*P* < 0.05, ##*P* < 0.01, ###*P* < 0.001 vs. CUS.

Masson's trichrome staining was used to assess atrial fibrosis. *Figure 8A* and *B* displayed the representative images of Masson staining and qualitative results, respectively. Atrial fibrosis was significantly increased in CUS rats (8.79 ± 0.54%) vs. CTL rats (3.45 ± 0.38%). The protein expression of fibrosis-related indicators including collage I, collage III, α-SMA, and TGFβ-1 were significantly increased (*Figure 8C*–*F*), which confirmed a higher level of atrial fibrosis in CUS rats. The increase in atrial fibrosis was prevented by BBG co-administration in CUS evidenced by the significant reduction of collagen deposition in Masson staining and lower expression of fibrosis-related indicators (*Figure 8A*–*F*).

### P2X7R inhibition prevented the down-regulations of ion channels and connexins in chronic unpredictable stress-induced rats

**Figure 9:**
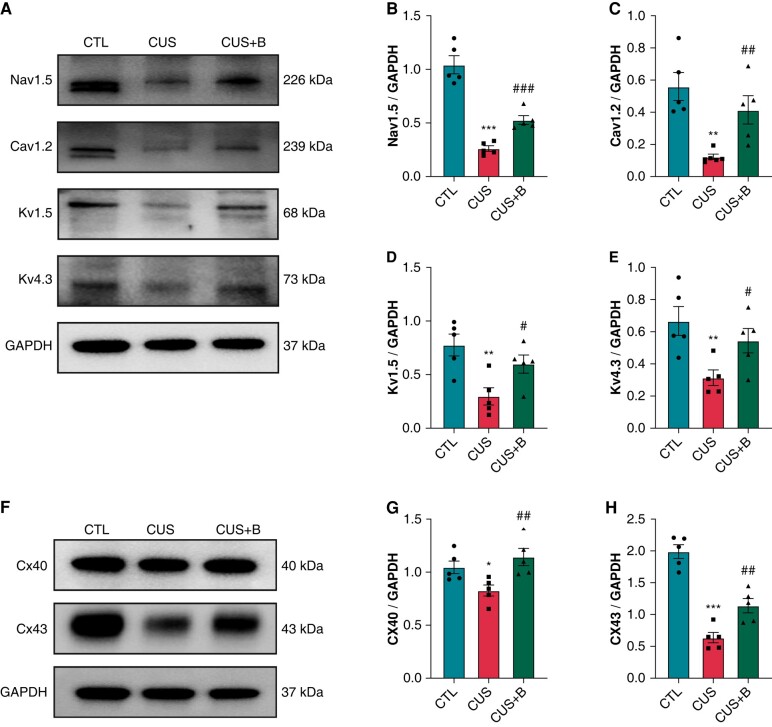
Protein expression of ion channels and connexins in CUS-induced rats. (*A*–*E*) Immunoblotting and expression ratio of Nav1.5, Cav1.2, Kv1.5, and Kv4.3, respectively. *n* = 5 per group. (*F*–*H*) Immunoblotting and expression ratio of Cx40 and Cx43, respectively. *n* = 5 per group. *P < 0.05, **P < 0.01, ***P < 0.001 vs. CTL; #*P* < 0.05, ##*P* < 0.01, ###*P* < 0.001 vs. CUS.

*Figure 9A*–*E* showed that the protein expression levels of Nav1.5, Cav.1.2, Kv1.5, and Kv4.3 were significantly reduced in CUS rats compared with the CTL rats, which indicated that abnormalities in ion channels were involved in CUS-related AF. BBG significantly prevented the down-regulations of these ion channels in CUS + B rats. In addition, both connexin (Cx) 40 and Cx43 showed significant decreases in CUS rats compared to the CTL rats, whereas P2X7R inhibition prevented the down-regulations (*Figure 9F*–*H*).

### P2X7R inhibition mitigated inflammation in chronic unpredictable stress-induced rats

**Figure 10:**
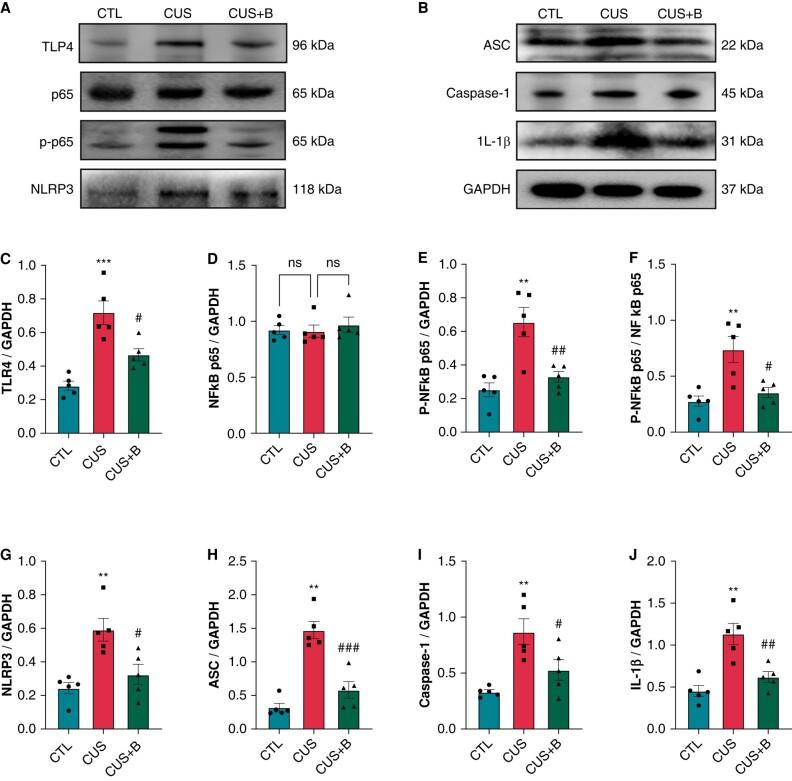
Expression of TLR4/NF-κB/NLRP3 pathway-related indicators in CUS-induced rats. (*A*–*E*) Immunoblotting and expression ratio of TLR4, p65, p-p65, NLRP3, ASC, Caspase-1, and IL-1β, respectively. *n* = 5 per group. **P < 0.01, ***P < 0.001 vs. CTL; #*P* < 0.05, ##*P* < 0.01, ###*P* < 0.001 vs. CUS.

NF-κB signalling pathway, an inducible transcription factor involved in the regulation of immune and inflammatory responses, may interrelate with NLRP3 inflammasome and can be activated after toll-like receptor (TLR) 4 stimulation. In this study, the protein expression of TLR4, P-NF-κB P65, NF-κB-P65/P-NF-κB P65, NLRP3, ASC, caspase-1, and IL-1β was significantly increased in CUS rats compared to CTL rats; BBG significantly prevented the alterations in CUS + B rats (*Figure 10A*–*J*).

It can also be observed that BBG alone did not change depressive-like behaviours, atrial electrophysiological parameters, and ECG indicators in CTL + B rats compared to CTL rats (*Figures 3*, *5*, and *6*). We further clarified that BBG treatment did not alter GAP43 and TH expression, atrial fibrosis, ion channels and connexins expression, and P2X7R/NLRP3/IL-1β pathway in normal rats (see Supplementary material online, *Figures S4* and *S5*).

In addition, 1 week after CUS induction did not promote depressive-like behaviours, AF inducibility, atrial fibrosis, and the activation of the P2X7R/NLRP3/IL-1β pathway compared to the controls (see Supplementary material online, *Figure S6*). These data suggested that an earlier time point after induction of CUS (on Day 7) did not activate NLRP3 inflammasome, and was not associated with atrial fibrosis or AF inducibility, whereas the activated NLRP3 inflammasome after 4-week CUS induction was associated with the increased atrial fibrosis and susceptibility to AF. Therefore, we deduced that CUS induction for a limited period did not activate NLRP3 inflammasome or increase AF inducibility, but the CUS-induced depression for a longer period activated NLRP3 and subsequently promoted atrial fibrosis and AF.

### P2X7R deficiency improved depressive-like behaviours and ameliorated atrial fibrillation inducibility in chronic unpredictable stress-induced mice

**Figure 11:**
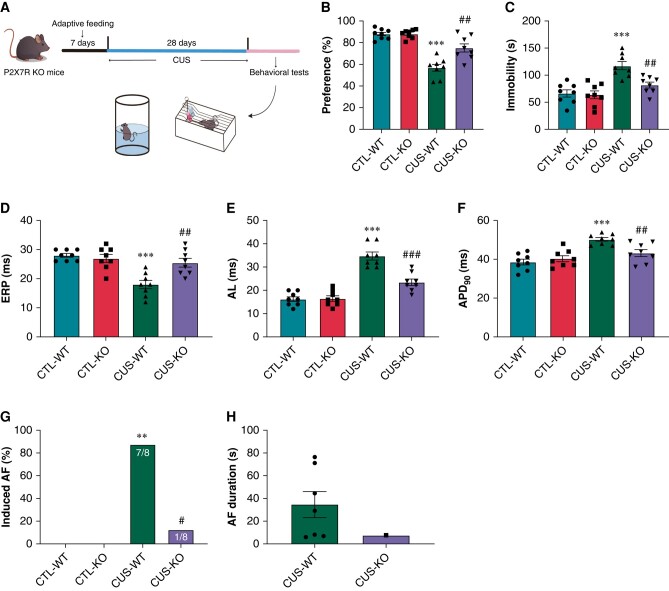
Depressive-like behaviours and electrophysiological phenotypes in CUS-induced mice. (*A*) Schematic diagram of the experimental procedure. (*B*) Sucrose preference in the SPT. *n* = 8 per group. (*C*) Immobility in the FST. *n* = 8 per group. (*D*–*F*) ERP, AL, and APD_90_, respectively. *n* = 8 per group. (*F*) Inducibility of AF. *n* = 8 per group. (*G*) Duration of AF. *n* = 7 in CUS-WT and *n* = 1 in CUS + KO. **P < 0.01, ***P < 0.001 vs. CTL-WT; #*P* < 0.05, ##*P* < 0.01, ###*P* < 0.001 vs. CUS-WT. SPT, sucrose preference test; FST, forced swimming test; ERP, effective refractory period; AL, atrial latency; APD_90_, action potential duration at 90% repolarization; AF, atrial fibrillation.

The above experiments indicated a non-negligible role of P2X7R in LPS or CUS-induced AF in rats. P2X7R-KO mice were used to further investigate the role of P2X7R in CUS-related AF. CUS was applied to induce depression in mice. *Figure 11A* briefly depicted the paradigm of the CUS-induced depression model in mice. CUS-WT mice reduced sucrose preference (*P* < 0.001, *Figure 11B*) in the SPT and increased immobility (*P* < 0.001; *Figure 11C*) in the FPT compared to CTL-WT mice, which was consistent with the results in CUS-induced rats. A similar trend of improvement in depressive-like behaviours was also observed in CUS-KO mice compared to CUS-WT mice (*Figure 11B* and *C*; all *P* < 0.01).

Similar to the results in CUS-induced rats, CUS-WT mice shortened ERP, and prolonged AL and APD_90_ compared to CTL-WT mice (all *P* < 0.001); P2X7R-KO significantly prevented these alterations in CUS-KO mice (*Figure 11D*–*F*). AF was induced in CUS-WT mice (seven of eight; 87.5%) and CUS-KO mice (one of eight; 12.5%); AF duration in CUS-WT mice tended to be longer compared to CUS-KO mice (*Figure 11G* and *H*).

### P2X7R deficiency prevented the abnormalities in P-wave parameters in chronic unpredictable stress-induced mice

**Figure 12:**
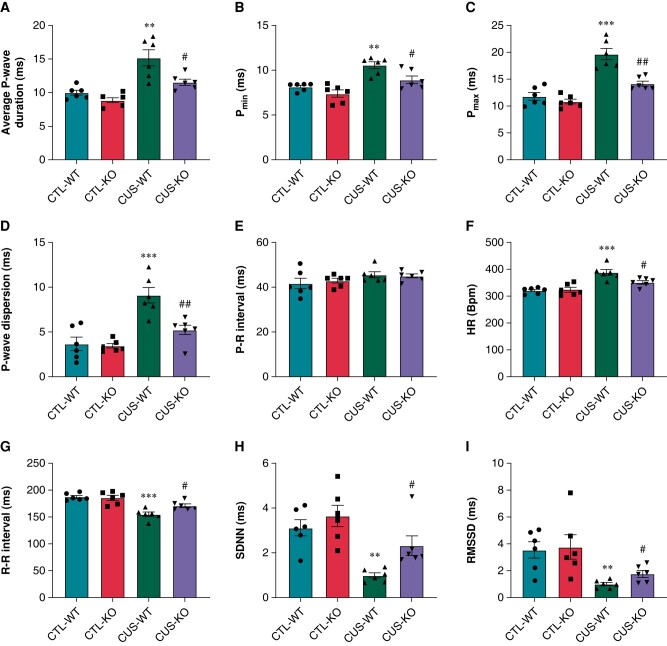
ECG and HRV parameters in CUS-induced mice. (*A*–*E*) Average P-average duration, Pmin, Pmax, P-wave dispersion, and P–R interval, respectively. *n* = 6 per group. (*F*–*I*) HR, R–R interval, SDNN, and RMSSD, respectively. *n* = 6 per group. **P < 0.01, ***P < 0.001 vs. CTL-WT; #*P* < 0.05, ##*P* < 0.01 vs. CUS-WT. HRV, heart rate variability.

As *Figure 12A*–*D* indicated, P-wave indicators including average P-wave duration, Pmin, Pmax, and P-wave dispersion, were significantly prolonged in CUS-WT mice compared to CTL-WT mice. Although no significant difference was observed, the P–R interval tended to be prolonged in CUS-WT mice (*Figure 12E*). P2X7R-KO prevented the prolongations of these ECG parameters (*Figure 12A*–*E*).

### P2X7R deficiency mitigated cardiac autonomic dysfunction and atrial fibrosis in chronic unpredictable stress-induced mice

**Figure 13:**
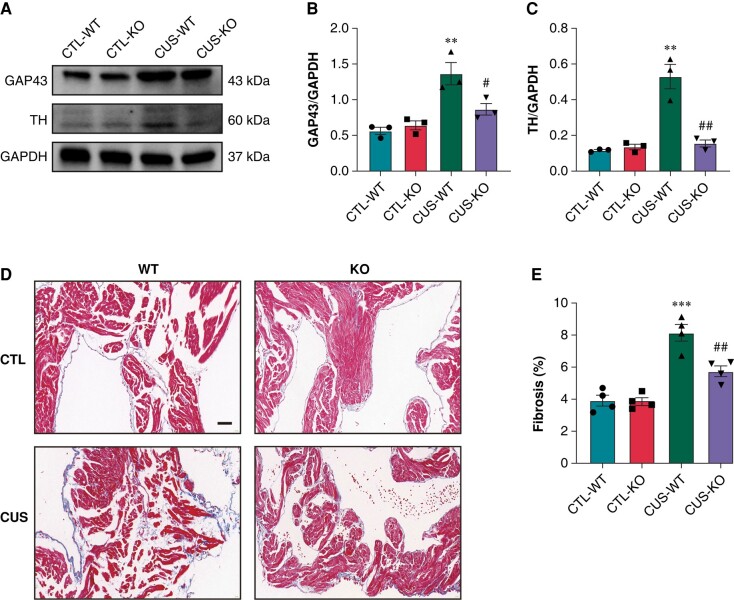
Expression of GAP43 and TH, and atrial fibrosis in CUS-induced mice. (*A*–*C*) Immunoblotting and expression ratio of GAP43 and TH, respectively. *n* = 3 per group. (*D*–*E*) Representative images of Masson staining in atria and quantification of the fibrotic area, respectively. *n* = 4 per group. Scale bar: 50 μm. **P < 0.01, ***P < 0.001 vs. CTL-WT; #*P* < 0.05, ##*P* < 0.01 vs. CUS-WT.

Increased HR and reduced R–R interval, SDNN, and RMSSD were found in CUS-WT mice compared to CTL-WT mice (*Figure 12F*–*I*). CUS-WT mice significantly up-regulated the protein expression of GAP43 and TH (*Figure 13A*–*C*) and promoted atrial fibrosis (*Figure 13D* and *E*). P2X7R-KO significantly mitigated these alterations in CUS-KO mice.

### P2X7R deficiency prevented the down-regulations of ion channels and connexins and the activation of NLRP3 inflammasome in chronic unpredictable stress-induced mice

**Figure 14:**
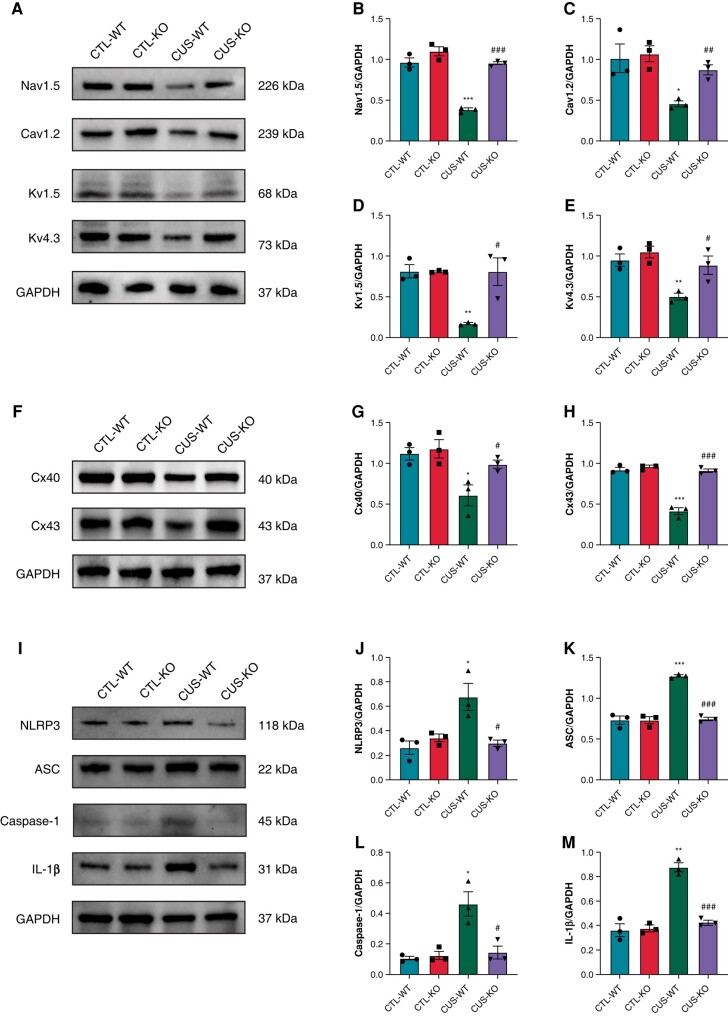
Protein expression of ion channels, connexins, and NLRP3 inflammasome-related indicators in CUS-induced mice. (*A*–*E*) Immunoblotting and expression ratio of Nav1.5, Cav1.2, Kv1.5, and Kv4.3, respectively. *n* = 3 per group. (*F*–*H*) Immunoblotting and expression ratio of Cx40 and Cx43, respectively. *n* = 3 per group. (*I*–*M*) Immunoblotting and expression ratio of NLRP3, ASC, Caspase-1, and IL-1β, respectively. *n* = 3 per group. *P < 0.05, **P < 0.01, ***P < 0.001 vs. CTL-WT; #*P* < 0.05, ##*P* < 0.01, ###*P* < 0.001 vs. CUS-WT.

As shown in *Figure 14A*–*F*, CUS caused significant reductions in ion channel expression including Nav1.5, Cav1.2, Kv1.5, and Kv4.3, and down-regulations of Cx40 and Cx43 compared to CTL-WT mice. P2X7R-KO significantly prevented the down-regulations of ion channels and connexins. Activated NLRP3 inflammasome and increased IL-1β expression were found in CUS-WT mice, whereas P2X7R-KO prevented NLRP3 inflammasome activation and the expression of IL-1β (*Figure 14I*–*M*).

## Discussion

Our results mainly indicated that pharmacological inhibition or genetic deficiency of P2X7R prevented LPS or CUS-induced AF. The expression of P2X7R was significantly up-regulated in LPS- and CUS-induced depression. P2X7R inhibition or deficiency prevented CUS-induced abnormalities in P-wave parameters (prolonged average P-wave duration, Pmax, and P-wave dispersion), autonomic dysfunction (depressed vagal tone and increased sympathetic activity), atrial fibrosis, and down-regulation of ion channels (Nav1.5, Cav1.2, and Kv1.5, and Kv4.3) and connexins (Cx40 and Cx43). Mechanistically, P2X7R might promote atrial remodelling via the TLR4/NF-κB/NLRP3 pathway.

Our previous studies indicated that a shortening of ERP was associated with an increased incidence of AF in rats.^23,24^ Reduced ventricular ERP was also observed in CUS-induced depression in rats, which was associated with the increased risk of VA.^13^ AL, mainly due to activation delay, is inversely proportional to the conduction velocity (CV) and has been implicated in facilitating re-entrant AF.^25^ Greater atrial AL was also found in rats with MI, which was correlated with an increased risk of AF.^24^ The refractory period, primarily driven by APD, is one of the main functional determinants of AF. Excessive APD prolongation promotes early after-depolarizations, which are proposed as important triggers of arrhythmogenesis. In addition, a reduction in the ERP and an increase in the APD have been recognized as two conceptual mechanisms for AF. Although shortened or unchanged APD was found in AF subjects, the prolongation of APD was also observed in AF-promoted animal models.^26,27^ It has also been proposed that disruptions in these electrophysiological indicators such as reduced CV, prolonged or abbreviated APD, or shortened ERP can cause atrial arrhythmias.^28^ In this study, CUS led to the shortening of ERP, the prolongations of AL and APD, and the increased AF inducibility, which was consistent in our previous work.^29^ whereas pharmacological inhibition using the P2X7R antagonist BBG or genetic P2X7R deficiency prevented the aforementioned electrophysiological phenomenon.

P-wave parameters were used to detect atrial conduction. In the present study, the average P-wave duration, Pmin, Pmax, and P-wave dispersion were significantly prolonged in CUS-induced rats and mice, whereas P2X7R antagonist or knockout could prevent these P-wave parameters. Prolonged P-wave duration has been proposed to be associated with increased AF risk and a recognized predictor of AF.^30^ It should be noted that Pmax was correlated with atrial conduction times and was associated with long-term AF risk, which may undertake an electrocardiographic endophenotype for AF.^30,31^ Although both Pmin and Pmax were increased in CUS, the overall result was the prolongation of P-wave dispersion, indicating that the contribution of the Pmax increase overcame that of the prolongation in the Pmin. P-wave dispersion was validated as an independent predictor of interatrial and intra-left atrial conduction times, and its prolongation was a significant predictor of AF.^32,33^ In summary, the prolongation of P-wave parameters represented a decrease in CV within the atria of CUS-induced rats and mice, which at least partly explained the possible substrate for the atrial arrhythmia caused by depression.

Dysfunction of ANS can occur in depression.^2^ For instance, stress can cause activation of the sympathetic nervous system and inhibition of the parasympathetic nervous system.^34^ HRV is commonly used to assess cardiac autonomic activity. Previous work indicated a reduction in HRV in individuals with psychiatric disorders, which may explain the increased risk of CVD.^35^ More importantly, our previous work has revealed that CUS causes lower HRV in rats than in controls.^13^ Decreases in mean RR, SDNN, and RMSSD and increases in HR together mainly represent a decrease in parasympathetic activity. In parallel, increases in LF and LF/HF ratio, and the decrease in HF elucidate the augment in sympathetic nerve activity and/or reduction in parasympathetic activity. Our results showed that CUS reduced mean RR, SDNN, RMSSD and HF, and increased LF and LF/HF in rats, revealing ANS disorders in CUS-induced depression. Previous studies have shown that ANS dysfunction provides substrates for atrial arrhythmias, and modulation of ANS activity has been recognized as a promising strategy for the treatment of AF.^36^ In particular, we discovered a significant increase in the levels of TH and GAP43, which revealed a higher degree of nerve growth and confirmed augmented sympathetic control in CUS-induced rats. The P2X7R inhibition not only improved HRV but also prevented the up-regulation of TH and GAP43, which further confirmed the effect of P2X7R on the improvement of autonomic function. Similarly, reduced HRV and increased expression of GAP43 and TH were also found in CUS-induced mice, whereas P2X7R-KO significantly mitigated these indicators, validating the dysfunction of ANS in depression and the protective effects of P2X7R in regulating ANS.

Cav1.2 is one of the transmembrane voltage-gated L-type calcium channels mediating Ca^2+^ flow. The mRNA expression of auxiliary subunits of atrial L-type Ca^2+^ channel was significantly reduced in patients with chronic AF,^37^ and down-regulation of L-type Ca^2+^ currents (I_Ca-L_) is associated with an increased risk of AF.^38^ The cardiac sodium channel Nav1.5, encoded by the SCN5A gene, is the basis for the rapid rise in action potential. Cumulative data suggested a strong link between AF and SCN5A mutations, and the association of loss-of-function mutations in SCN5A with AF has been reported.^39^ The voltage-gated potassium channel Kv1.5, conducting the ultrarapid delayed rectifier current (I_Kur_), is the main repolarizing current in the atria. Attenuation of I_Kur_ prolongs APD and thus provokes atrial arrhythmia. The protein expression of Kv1.5 was observed to be down-regulated in patients with AF.^40^ Kv4.3 (KCND3), belonging to the Kv4 subfamily, partly encodes the cardiac fast transient outward potassium current (Ito) channel. Ito is an essential component of the early phase of action potential repolarization and plateau potential and can influence the activation of voltage-gated Ca^2+^ channels, thus balancing the inward and outward currents.^41^ Similar to Kv1.5, the expression of Kv4.3 was also down-regulated in AF patients.^40^ In the present study, CUS led to the down-regulations of Nav1.5, Cav1.2, Kv1.5, and Kv4.3 in atria, which may contribute to the occurrence of AF. P2X7R inhibition or deficiency prevented the down-regulations of these ion channels, representing a protective effect on cardiac electrophysiology.

Myocardial fibrosis is characterized by abnormal proliferation and differentiation of fibroblasts with pathological synthesis and deposition of extracellular matrix (ECM) proteins. Fibroblasts and myofibroblasts can also influence action potential conduction by forming gap junctions with cardiac myocytes.^42^ Although myocardial fibrosis, whether reactive or reparative, maintains the integrity of the heart, pathological deposition of ECM alters the ultrastructure of the heart and eventually impairs the cardiac conduction system.^43^ Inflammatory markers such as TGF-β1 have been reported to be elevated in patients with AF and promote atrial fibrosis.^43^ Whilst the exact mechanism is unclear, gap junction proteins are responsible for the electrical coupling between adjacent cardiomyocytes, and their abundance and spatial distribution correlate with susceptibility to AF. The human atrium chiefly expresses Cx40 and Cx43, and abnormalities in Cx40 levels and distribution were prevalent in AF.^44^ Previous pre-clinical work has also shown that Cx40-KO increases the susceptibility to atrial arrhythmias in mice.^45^ Similarly, Cx43 plays a crucial role in cardiac development and down-regulation of Cx43 triggers sympathetic AF.^46^ These studies all highlight the contribution of the abundance and distribution of gap junctions to the maintenance of normal electrophysiology. Our data showed increased myocardial fibrosis and decreased expression levels of Cx43 and Cx40 in CUS, whereas P2X7R inhibition or deficiency was effective in preventing these abnormalities in this model.

Inflammatory response is a pivotal component in the development of both depression and AF. Stimulation of TLR4 activates its downstream effector NF-κB (p50/p65) and can promote the priming of NLRP3 inflammasome, evoking the production of inflammatory cytokines such as TNF-α and IL-1β. NLRP3 inflammasome has been recognized to be an important contributor to the development of both depression^47^ and AF.^19^ P2X7R, one of the major activator of NLRP3, is also a potent inducer of activation in microglia and macrophage, and proliferation in the neuroinflammatory response. Although pre-clinical studies have shown that activation of the P2X7R-NLRP3-IL-1β pathway induces and maintains depressive-like behaviours,^48^ the mechanisms underlying AF in depression have not been specifically explored. A previous study has indicated that P2X7R is up-regulated in infarcted hearts and P2X7R inhibition inhibits the activation of NF-κB and suppresses sympathetic activation in MI.^49^ A latest study pointed out that the ATP/P2X7 axis played an instrumental role in myocardial inflammation and hypertrophy, in which heart–brain interaction evoked sympathetic efferent nerves to release extracellular ATP, subsequently inducing hypertrophic lesions in cardiomyocytes.^50^ However, to our knowledge, we are the first to investigate the role of P2X7R in AF in depression models. In this study, NLRP3 inflammasome was activated in atria in both LPS- and CUS-induced depression. TLR4 and NF-κB, important regulators of NLRP3-complex priming, were also increased, which further confirmed the inflammation condition in atria of depression. P2X7R inhibition or deficiency prevented NLRP3 inflammasome activation and the production of IL-1β, suggesting a potential mechanism for P2X7R in mediating atrial remodelling in depression.

## Conclusion

In this study, CUS caused atrial electrical remodelling, autonomic dysfunction, atrial fibrosis, as well as ion-channel and connexins abnormalities, resulting in a predisposition to inducible AF. P2X7R inhibition prevented atrial remodelling at least by inhibiting the TLR4-NF-κB-NLRP3 pathway. Our results, using rat and mouse models of depression, suggest that P2X7R is involved in the pathogenesis of AF following depression and imply that targeting P2X7R could be a promising target for AF in depression.

## Limitations

This study has several limitations. First, despite that the increased AF inducibility was found in both LPS- and CUS-induced depression models in rats, no further experiments were performed to explore the mechanisms of LPS-induced depression. However, CUS is a classic and the most commonly used rodent model of depression, which has been investigated in detail in this study. Secondly, we only measured the protein expression of ion channels, but did not perform patch clamp experiments to detect ion channel currents, which should be addressed in future studies. Thirdly, it remains to be investigated how much can atrial electrophysiological changes in depression models be linked directly to P2X7R instead of other ion channels such as Cav1.2, Kv4.2, and Kv1.5 given that P2X7R itself is also an ion channel, which needs further exploration. Finally, although the TLR4/NF-κB/NLRP3 pathway was identified as a potential mechanism in this study, P2X7R inhibition may prevent electrophysiology through other mechanisms.

## Supplementary Material

euae022_Supplementary_Data

## Data Availability

The data underlying this article will be shared on reasonable request to the corresponding author.
