# Supplementary material for: P2X7 receptor inhibition prevents atrial fibrillation in rodent models of depression
Source: Europace. 2024 Jan 23;26(2):euae022. doi: 10.1093/europace/euae022 (PMC10873709; doi:10.1093/europace/euae022)
Supplement: euae022_Supplementary_Data [file euae022_supplementary_data.zip › Supplementary_Legends.docx]

**Figure. S1** Immunofluorescence of P2X7R in LPS-induced depression. (A-B) Representative images of double-immunofluorescent labeling of P2X7R and Cardiac Troponin T, and the mean fluorescence intensity of P2X7R, respectively. n=4 per group. (C) Negative control was performed using specific IgG instead of the P2X7R antibody. Scale bar: 50 um. ***P < 0.001 vs. CTL; ##P < 0.01 vs. LPS. AU, arbitrary units.

**Figure. S2** Representative ECG traces for analysis.

**Figure. S3** Immunofluorescence of GAP43 and TH in CUS-induced rats. (A-B) Representative immunofluorescent images and the mean fluorescence intensity of GAP43 and TH, respectively. n=4 per group. (C) Negative control was performed using specific IgG instead of the GAP43 or TH antibodies. Scale bar: 50 um. **P < 0.01, ***P < 0.001 vs. CTL; #P < 0.05 vs. CUS. AU, arbitrary units.

**Figure. S4** Expression of GAP43 and TH, atrial fibrosis, and ion channel expression in BBG treatment rats. (A-C) Immunoblotting and expression ratio of GAP43 and TH, respectively. n=4 per group. (D-E) Representative images of Masson staining in atria and quantification of the fibrotic area, respectively. n=4 per group. Scale bar: 100 um. (F-J) Immunoblotting and expression ratio of Nav1.5, Cav1.2, Kv1.5, and Kv4.3, respectively. n=4 per group. NS, no significant difference.

**Figure. S5** Protein expression of connexins and NLRP3 inflammasome-related indicators in BBG treatment rats. (A-C) Immunoblotting and expression ratio of Cx40 and Cx43, respectively. n=4 per group. (D-I) Immunoblotting and expression ratio of P2X7R, NLRP3, ASC, Caspase-1, and IL-1β, respectively. n=4 per group. NS, no significant difference.

**Figure. S6** Depressive-like behaviors, AF inducibility, atrial fibrosis, and NLRP3 inflammasome-related indicators after 1-week CUS induction in rats. (A) Sucrose preference in the SPT. n=6 per group. (B) Immobility in the FST. n=6 per group. (C) Inducibility of AF. n=6 per group. (D-E) Representative images of Masson staining in atria and quantification of the fibrotic area, respectively. n=4 per group. Scale bar: 100 um. (F-K) Immunoblotting and expression ratio of P2X7R, NLRP3, ASC, Caspase-1, and IL-1β, respectively. n=4 per group. NS, no significant difference.
