# Supplementary material for: P2X7 receptor inhibition prevents atrial fibrillation in rodent models of depression
Source: Europace. 2024 Jan 23;26(2):euae022. doi: 10.1093/europace/euae022 (PMC10873709; doi:10.1093/europace/euae022)
Supplement: euae022_Supplementary_Data [file euae022_supplementary_data.zip › Table. S1.docx]

**Table. S1** Details of the stressors and duration of chronic unpredictable stress paradigm.

|  | **Week1** | **Week2** | **Week3** | **Week4** |
| --- | --- | --- | --- | --- |
| **Mon** | cage tilted at 45°C | tail pinched | ice water swimming | behavioral restriction |
| **Tues** | moist bedding | cage shaken | tail pinched | moist bedding |
| **Weds** | behavioral restriction | overnight illumination | cage shaken | water deprivation |
| **Thurs** | ice water swimming | predator sounds | cage tilted at 45°C | noise |
| **Fri** | fasting | noise | overnight illumination | hot water swimming |
| **Sat** | hot water swimming | moist bedding | fasting | overnight illumination |
| **Sun** | water deprivation | behavioral restriction | predator sounds | cage tilted at 45°C |
