# Supplementary material for: P2X7 receptor inhibition prevents atrial fibrillation in rodent models of depression
Source: Europace. 2024 Jan 23;26(2):euae022. doi: 10.1093/europace/euae022 (PMC10873709; doi:10.1093/europace/euae022)
Supplement: euae022_Supplementary_Data [file euae022_supplementary_data.zip › Table. S2.docx]

**Table. S2** HF and LF bands for analysis of HRV.

| Preset | LF (Hz) | HF (Hz) |
| --- | --- | --- |
| Rat | 0.20-0.75 | 0.75-2.50 |
| Mouse | 0.15-1.50 | 1.50-5.00 |

HF, high frequency; LF, low frequency; HRV, Heart rate variability.
