# Supplementary material for: P2X7 receptor inhibition prevents atrial fibrillation in rodent models of depression
Source: Europace. 2024 Jan 23;26(2):euae022. doi: 10.1093/europace/euae022 (PMC10873709; doi:10.1093/europace/euae022)
Supplement: euae022_Supplementary_Data [file euae022_supplementary_data.zip › Table. S3.docx]

**Table S3**

Antibodies used for Western blot experiments.

| Target | Antibody manufacturer | Dilution |
| --- | --- | --- |
| P2X7R | Santa Cruz | 1:100 |
| Nav1.5 | Affinity | 1:1000 |
| Cav1.2 | Proteintech | 1:1000 |
| Kv4.3 | Affinity | 1:1000 |
| Kv1.5 | Immunoway | 1:1000 |
| Cx40 | Affinity | 1:1000 |
| Cx43 | Cell Signaling Technology | 1:1000 |
| Collage-I | Affinity | 1:1000 |
| Collage-III | Affinity | 1:1000 |
| TGF-β1 | Affinity | 1:1000 |
| TH | Affinity | 1:1000 |
| GAP43 | Affinity | 1:1000 |
| TLR4 | Affinity | 1:1000 |
| p65 | Affinity | 1:1000 |
| p-p65 | Affinity | 1:1000 |
| NLRP3 | Affinity | 1:1000 |
| ASC | Affinity | 1:1000 |
| Caspase-1 | Affinity | 1:1000 |
| IL-1β | Affinity | 1:1000 |
| GAPDH | Affinity | 1:3000 |
