# Supplementary material for: P2X7 receptor inhibition prevents atrial fibrillation in rodent models of depression
Source: Europace. 2024 Jan 23;26(2):euae022. doi: 10.1093/europace/euae022 (PMC10873709; doi:10.1093/europace/euae022)
Supplement: euae022_Supplementary_Data [file euae022_supplementary_data.zip › Detailed Methods.docx]

**2. Methods**

**2.1 Animals**

Healthy male Sprague-Dawley (SD) rats (180-220 g), wild-type (WT) C57BL/6 (8-12 weeks) and P2X7R-knockout (KO) mice were housed under standard conditions (12-h light/dark cycle), allowing free access to food and water (unless indicated if the special environment was needed). P2X7R-KO mice were purchased from GemPharmatech (Nanjing, China). Animal studies were approved by the Animal Ethics Committee of the Tongren Hospital of Wuhan University (Wuhan Third Hospital; approval number: SY2020-042) and were conducted conformed to the NIH Guide for the Care and Use of Laboratory Animals.

**2.2 Models of depression**

**2.2.1 LPS-induced depression**

It has been found that there is a strong link between depression and inflammatory phenomena^1^. Lipopolysaccharide (LPS), a potent activator of the immune system, is widely used for inflammation-associated depression in rodents^2,3^. Rats were randomly grouped as follows: i) CTL group: saline; ii) LPS group: LPS; iii) LPS+B30 group: LPS + 30 mg/kg Brilliant Blue G (BBG; Sigma); iv) LPS+B50 group: LPS + BBG (50 mg/kg). LPS (Escherichia coli 055:B5, sigma) was solubilized in saline and injected (0.5 mg/kg) once daily for a total of 7 days^3^. BBG, widely used as a potent P2X7R antagonist, was solubilized in saline and administered at a dose of 30 or 50 mg/kg daily for 7 days. The dose of BBG was selected based on previous work^4^ and our preliminary results. LPS and BBG were injected intraperitoneally (i.p.). The subsequent experiments were conducted 24 hours after the establishment of LPS-induced depression.

**2.2.2 CUS-induced depression**

Chronic unpredictable stress (CUS) is the most effective and commonly used rodent model of depression in reflecting disease complexity^5^. CUS was carried out based on previously published studies with minor modifications^3,6,7^. In brief, after 7 days of adaptive feeding, rats or mice were exposed to one of the following stressors for 28 consecutive days: (1) cage tilted at 45°C for 24h, (2) moist bedding for 24h, (3) behavioral restriction for 2h, (4) ice water swimming at 4°C for 5min, (5) hot water swimming at 42°C for 5min, (6) fasting for 24 h, (7) water deprivation for 24 h, (8) tail pinched for 1 min (1 cm from the tail root), (9) cage shaken for 15 min, (10) overnight illumination, (11) predator sounds for 30 min, and (12) noise for 3 h. Table S1 showed the details of the stressors and duration of the CUS paradigm. The stressors were performed at random times during the day with the same stimulus not occurring consecutively to maximize the unpredictability of the stressor.

In experiments of CUS-induced rats, animals were randomly divided into four groups as follows: i) CTL group: saline; ii) CTL+B group: BBG (30 mg/kg); iii) CUS group: 4-week CUS + saline; and iv) CUS+B group: 4-week CUS + BBG (30 mg/kg). For CTL+B and CUS+B rats, BBG was given once (i.p.) daily for 28 days starting on day 1 of CUS construction. The CTL and the CUS rats were given equal amounts of saline, and the follow-up experiments were performed 24 h after the end of 28 days of the CUS protocol.

In experiments of CUS-induced mice, WT C57BL/6 and P2X7R-KO mice were randomly exposed to multiple stressors as above-mentioned for 4 weeks. Mice were randomly divided as follows: i) CTL-WT group: WT mice; ii) CTL-KO group: P2X7R-KO mice; iii) CUS-WT group: 4-week CUS in WT mice; and iv) CUS-KO group: 4-week CUS in P2X7R-KO mice. Control animals received no stressors. The subsequent experiments were conducted 24 hours after the establishment of CUS-induced depression in mice.

The 1-week CUS induction was also carried out in rats to establish a cause-effect relation between depression and AF.

**2.3 Behavioral tests**

**2.3.1 Sucrose preference test**

Sucrose preference test (SPT) was conducted to evaluate stress-induced anhedonia. Before the test, rats or mice were habituated for 48 hours to 1% sucrose and then received deprivation of water and food for 24 hours. Two identical bottles were prepared, one with 1% sucrose solution and the other with an equal amount of plain water, and the rats were allowed to drink freely for 1 hour. The sucrose preference rate was calculated as a percentage of the sucrose intake relative to the total liquid intake (sucrose intake + water intake).

**2.3.2 Forced swimming test**

Forced swimming test (FST) was performed to measure the immobility to assess behavior despair. Rats or mice were given 2 min to acclimatize to the new environment (a cylinder, height 50 cm, diameter 20 cm; 40 ± 1.5 cm water at 24 ± 0.5°C) in each experiment, and the time when rats or mice were immobile was recorded for the next 4 min.

**2.4 Body weight**

Animal body weight was measured and recorded after 4-week CUS in rats.

**2.5 Surface electrocardiogram**

Rats were anesthetized with pentobarbital sodium (40 mg/kg) and then implanted with a monitoring device. Electrocardiogram (ECG; lead II) was recorded continuously for 15 min and was analyzed manually by a blinded observer using the PowerLab system (4/35, AD Instruments, Australia). Manual measurements were performed as automatic measurements tend to underestimate the P wave duration and are subject to errors in cases of noisy tracings^8,9^.

**2.5.1 Electrocardiogram parameters**

The average P-wave duration, the shortest P-wave duration (Pmin), the longest P-wave duration (Pmax), P-wave dispersion, and P-R interval were manually calculated using LabChart 8.0 software. ECG morphology was assessed by a blinded observer from signal-averaged ECG recordings. Signal-averaged tracings of 4 consecutive beats were obtained, and at least 50 tracings per animal were used for calculation. P-wave duration and P-R interval were determined and measured according to the previous studies^9,10^. P-wave dispersion is obtained by subtracting the Pmin from the Pmax.

**2.5.2 Heart rate variability**

Heart rate variability (HRV) was measured in both time and frequency domain indicators according to the previous work^11-13^. Time domain indicators included heart rate (HR), mean RR interval, the standard deviation of normal RR intervals (SDNN), and the square root of the mean squared differences of successive RR intervals (RMSSD). Low frequency (LF), high frequency (HF), and LF /HF ratio belonged to frequency domain indicators. The Lomb Periodogram nonparametric method (time window: 5 min) was used for spectral analysis of frequency domain indicators of HRV. For analysis of frequency domain indicators, HF and LF bands were shown in Table S2.

**2.6 Atrial electrophysiological measurement**

Animals were anesthetized by intraperitoneal administration of sodium pentobarbital (40 mg/kg) and heparinized using sodium heparin (400 U). Hearts were isolated and were immediately perfused using the Langerdorff technique (AD Instruments, Dunedin, New Zealand) as described in our previous studies^7,11,12^. Tissue ECG and epicardial monophasic action potential (MAP) from the left atrial appendage (LAA) were recorded after a stabilization period of 10 minutes by perfusion at a constant flow before programmed electric stimulation. The MAP was measured using two custom-made Ag-AgCl electrodes (diameter: 0.25 mm and spacing: 0.5 mm) which were paired with platinum stimulating electrodes (diameter: 0.25 mm and spacing: 1 mm).

The S1-S2 program consisted of 8 consecutive basic stimuli S1 [pacing cycle length (PCL): 200 ms] and one preceding stimulus S2, and the PCL of S1-S2 was gradually shortened from 100 ms to 1 ms. The effective refractory period (ERP) was determined as the longest S1-S2 interval that failed to catch atrial activity. Atrial activation latency (AL) referred to the time required from the onset of each S2 to the maximum dV/dt during the action potential upstroke of each paced beat.

The S1-S1 stimulation procedure (PCL: 200 ms) was conducted with 10 stimuli. The action potential duration (APD) was measured at 90% repolarization (APD_90_). Atrial arrhythmias were induced by burst pacing at 50 Hz (pulse width 2 ms, 4x threshold) for 2 s, repeated no more than 6 times, and separated by 2-s intervals. Atrial tachycardia was defined as consecutive atrial extrasystoles with a cycle length shorter than that in sinus rhythm. A rapid irregular atrial rhythm with an irregular ventricular response lasting at least 2 s was considered AF, and a regular atrial tachyarrhythmia was determined as atrial flutter (AFL)^14^. The PowerLab system (AD Instruments) and LabChart 8.0 software were applied to record and analyze all the signals.

**2.7 Masson's trichrome staining**

The left atrium was extracted and fixed with 4% paraformaldehyde and embedded in paraffin. After cutting the paraffin into 5 μm sections, Masson's trichrome method was used to observe the distribution and deposition of myocardial collagen. ImageJ software was used to analyze the degree of fibrosis.

**2.8 Immunofluorescence**

Paraffin sections were washed 3 times for 5 min with PBS and then blocked for 1 h. Primary antibodies against P2X7R (diluted 1:200, Santa Cruz), GAP43 (diluted 1:200; Affinity), and tyrosine hydroxylase (TH, diluted 1:200; Affinity) were applied overnight at 4°C. Slides were then incubated with the secondary antibody. P2X7R antibody (diluted 1:200, Santa Cruz) and Cardiac troponin T antibody (diluted 1:100; Affinity) were employed for double immunofluorescent staining in atrial tissues.

**2.9 Western blot**

After extraction of total protein from atrial tissues and determination of its concentration, samples were run on 4%-12% sodium dodecyl sulfate-polyacrylamide gel electrophoresis and subsequently transferred to polyvinylidene difluoride membranes. After being blocked with the blocking solution, the membrane was incubated with the appropriate primary antibody and secondary antibodies. The primary antibodies were listed in Table S3.

**2.10 Statistical analysis**

Continuous variables were expressed as mean ± SEM. The distribution of data was assessed using the Shapiro-Wilk test. The unpaired two-tailed Student's t-test, Welch correction' t-test, or Mann-Whitney U test, was used for comparison between two groups when appropriate. Categorical variables were expressed as percentages. Atrial electrophysiological measurements were not performed in all subjects as the perfused hearts will not be used for other experiments such as histology or molecular measurements. Fisher’s exact test was used to analyze the AF inducibility in Figures 1E (n=8 per group), 5G (n=10 per group), and 11G (n=8 per group), and Figure. S6C (n=6 per group). Differences among more than 2 groups were compared using one-way ANOVA followed by Bonferroni’s post hoc test or Kruskal-Wallis test as appropriate. The p-value < 0.05 was regarded as statistically significant.

**References**

1. Zorrilla EP, Luborsky L, McKay JR, Rosenthal R, Houldin A, Tax A, et al. The relationship of depression and stressors to immunological assays: a meta-analytic review. *Brain Behav Immun.* 2001;15(3):199-226.

2. Adzic M, Djordjevic J, Mitic M, Brkic Z, Lukic I, Radojcic M. The contribution of hypothalamic neuroendocrine, neuroplastic and neuroinflammatory processes to lipopolysaccharide-induced depressive-like behaviour in female and male rats: Involvement of glucocorticoid receptor and C/EBP-beta. *Behav Brain Res.* 2015;291:130-139.

3. Song Q, Fan C, Wang P, Li Y, Yang M, Yu SY. Hippocampal CA1 betaCaMKII mediates neuroinflammatory responses via COX-2/PGE2 signaling pathways in depression. *J Neuroinflammation.* 2018;15(1):338.

4. Farooq RK, Tanti A, Ainouche S, Roger S, Belzung C, Camus V. A P2X7 receptor antagonist reverses behavioural alterations, microglial activation and neuroendocrine dysregulation in an unpredictable chronic mild stress (UCMS) model of depression in mice. *Psychoneuroendocrinology.* 2018;97:120-130.

5. Antoniuk S, Bijata M, Ponimaskin E, Wlodarczyk J. Chronic unpredictable mild stress for modeling depression in rodents: Meta-analysis of model reliability. *Neurosci Biobehav Rev.* 2019;99:101-116.

6. Iwata M, Ota KT, Li XY, Sakaue F, Li N, Dutheil S, et al. Psychological Stress Activates the Inflammasome via Release of Adenosine Triphosphate and Stimulation of the Purinergic Type 2X7 Receptor. *Biol Psychiatry.* 2016;80(1):12-22.

7. Ye T, Zhang C, Wu G, Wan W, Guo Y, Fo Y, et al. Pinocembrin Decreases Ventricular Fibrillation Susceptibility in a Rat Model of Depression. *Front Pharmacol.* 2020;11:547966.

8. Chen LY, Ribeiro ALP, Platonov PG, Cygankiewicz I, Soliman EZ, Gorenek B, et al. P Wave Parameters and Indices: A Critical Appraisal of Clinical Utility, Challenges, and Future Research-A Consensus Document Endorsed by the International Society of Electrocardiology and the International Society for Holter and Noninvasive Electrocardiology. *Circ Arrhythm Electrophysiol.* 2022;15(4):e010435.

9. Burke GM, Wang N, Blease S, Levy D, Magnani JW. Assessment of reproducibility--automated and digital caliper ECG measurement in the Framingham Heart Study. *J Electrocardiol.* 2014;47(3):288-293.

10. Magnani JW, Johnson VM, Sullivan LM, Gorodeski EZ, Schnabel RB, Lubitz SA, et al. P wave duration and risk of longitudinal atrial fibrillation in persons >/= 60 years old (from the Framingham Heart Study). *Am J Cardiol.* 2011;107(6):917-921 e911.

11. Ye T, Liu X, Qu C, Zhang C, Fo Y, Guo Y, et al. Chronic inhibition of the sigma-1 receptor exacerbates atrial fibrillation susceptibility in rats by promoting atrial remodeling. *Life Sci.* 2019;235:116837.

12. Ye T, Zhang C, Wu G, Wan W, Liang J, Liu X, et al. Pinocembrin attenuates autonomic dysfunction and atrial fibrillation susceptibility via inhibition of the NF-kappaB/TNF-alpha pathway in a rat model of myocardial infarction. *Int Immunopharmacol.* 2019;77:105926.

13. Shi S, Liu T, Wang D, Zhang Y, Liang J, Yang B, et al. Activation of N-methyl-d-aspartate receptors reduces heart rate variability and facilitates atrial fibrillation in rats. *Europace.* 2017;19(7):1237-1243.

14. Hiram R, Xiong F, Naud P, Xiao J, Sirois M, Tanguay JF, et al. The inflammation-resolution promoting molecule resolvin-D1 prevents atrial proarrhythmic remodelling in experimental right heart disease. *Cardiovasc Res.* 2021;117(7):1776-1789.
