# Supplementary figures and images for: P2X7 receptor inhibition prevents atrial fibrillation in rodent models of depression
Source: Europace. 2024 Jan 23;26(2):euae022. doi: 10.1093/europace/euae022 (PMC10873709; doi:10.1093/europace/euae022)

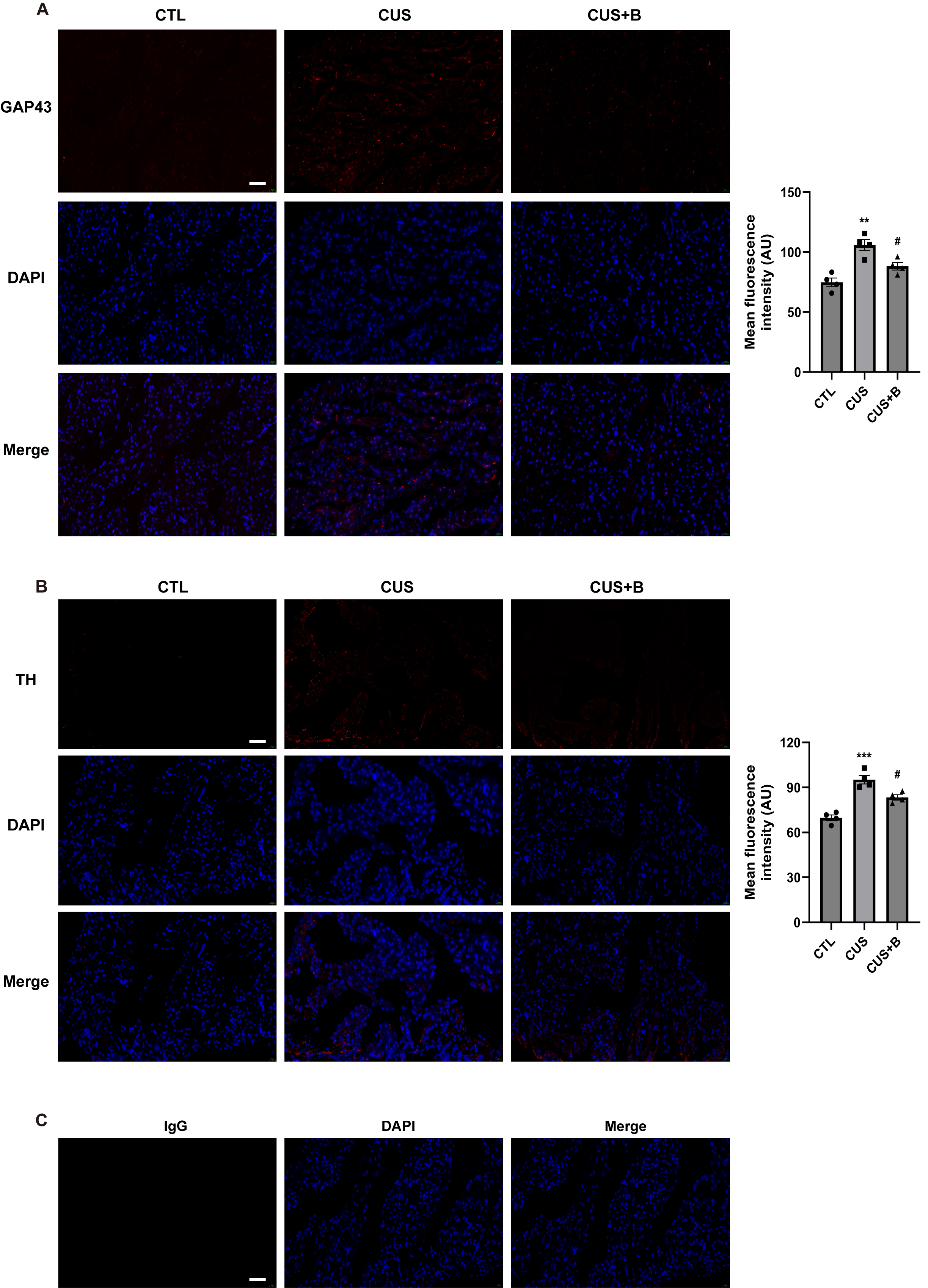

Supplement: euae022_Supplementary_Data [file euae022_supplementary_data.zip › Figure. S3.tif]

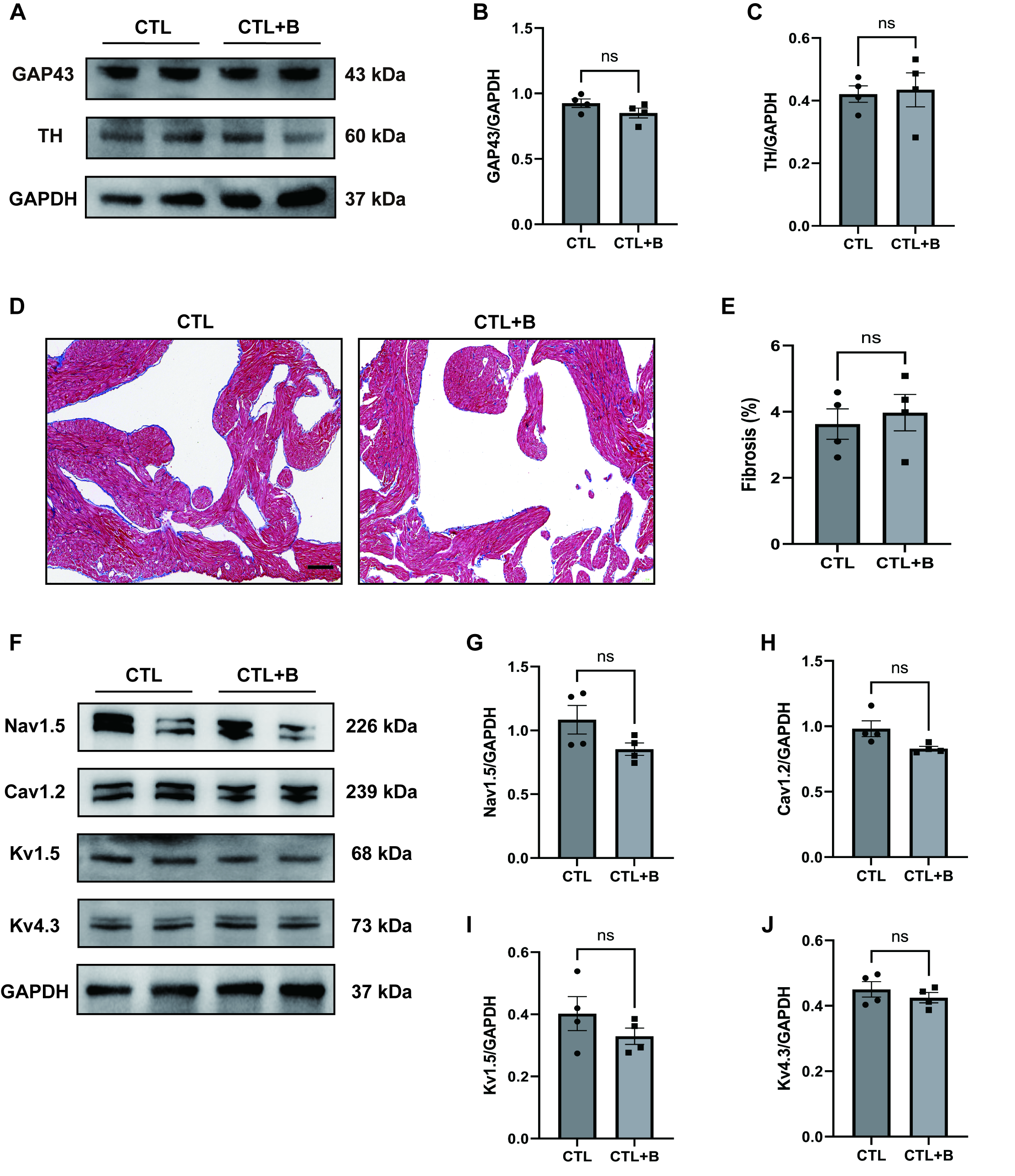

Supplement: euae022_Supplementary_Data [file euae022_supplementary_data.zip › Figure. S4.tif]

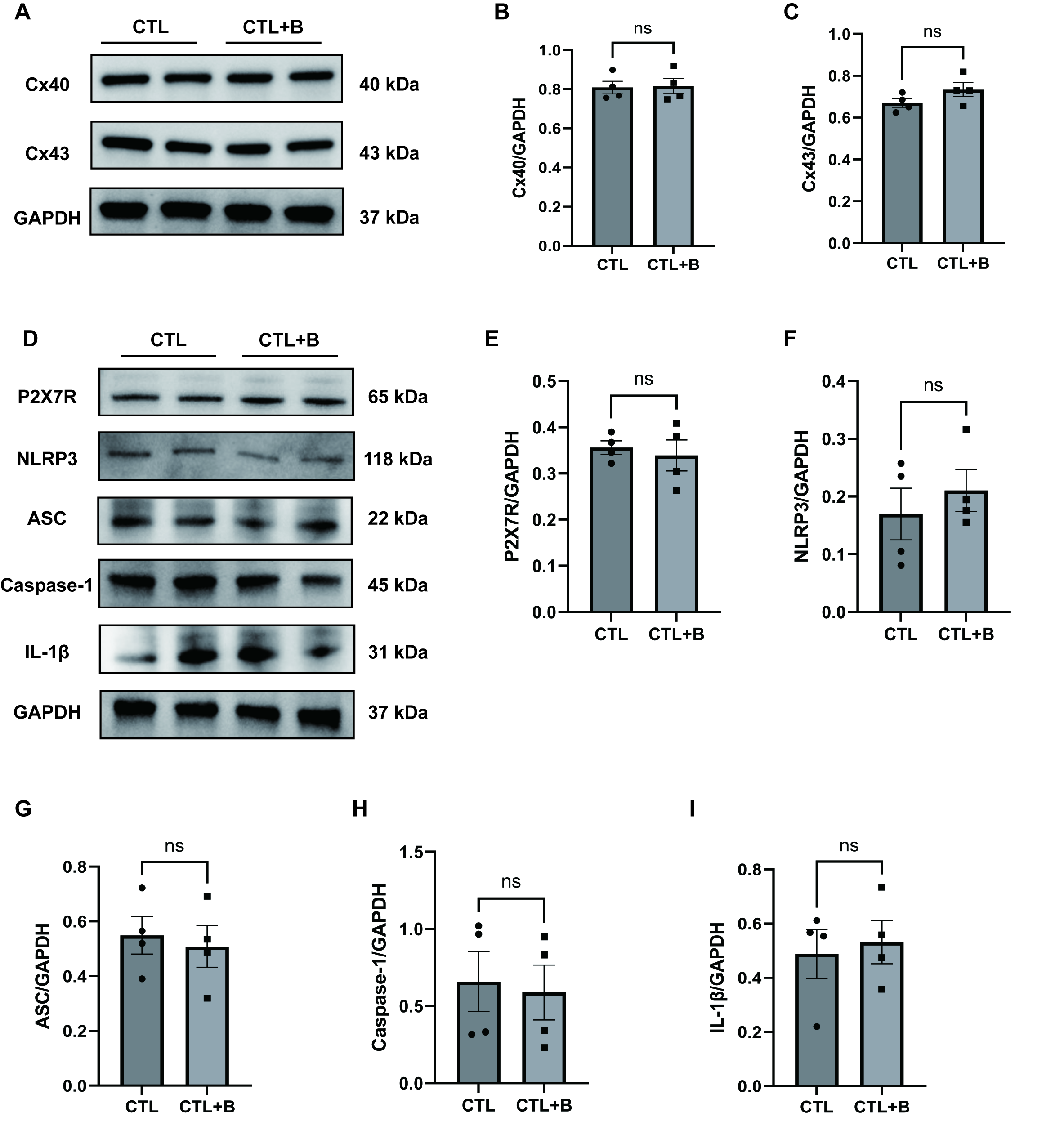

Supplement: euae022_Supplementary_Data [file euae022_supplementary_data.zip › Figure. S5.tif]

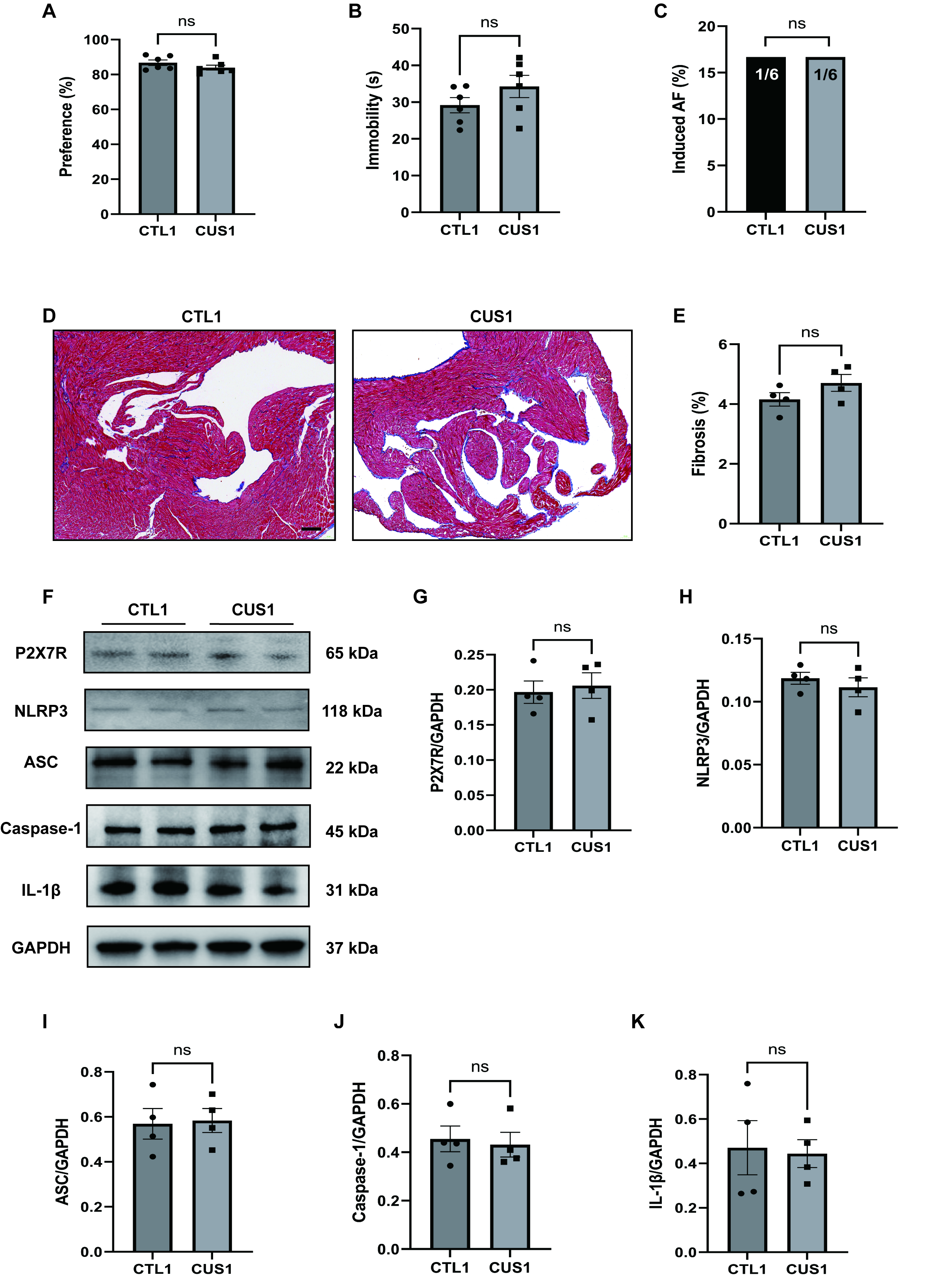

Supplement: euae022_Supplementary_Data [file euae022_supplementary_data.zip › Figure. S6.tif]

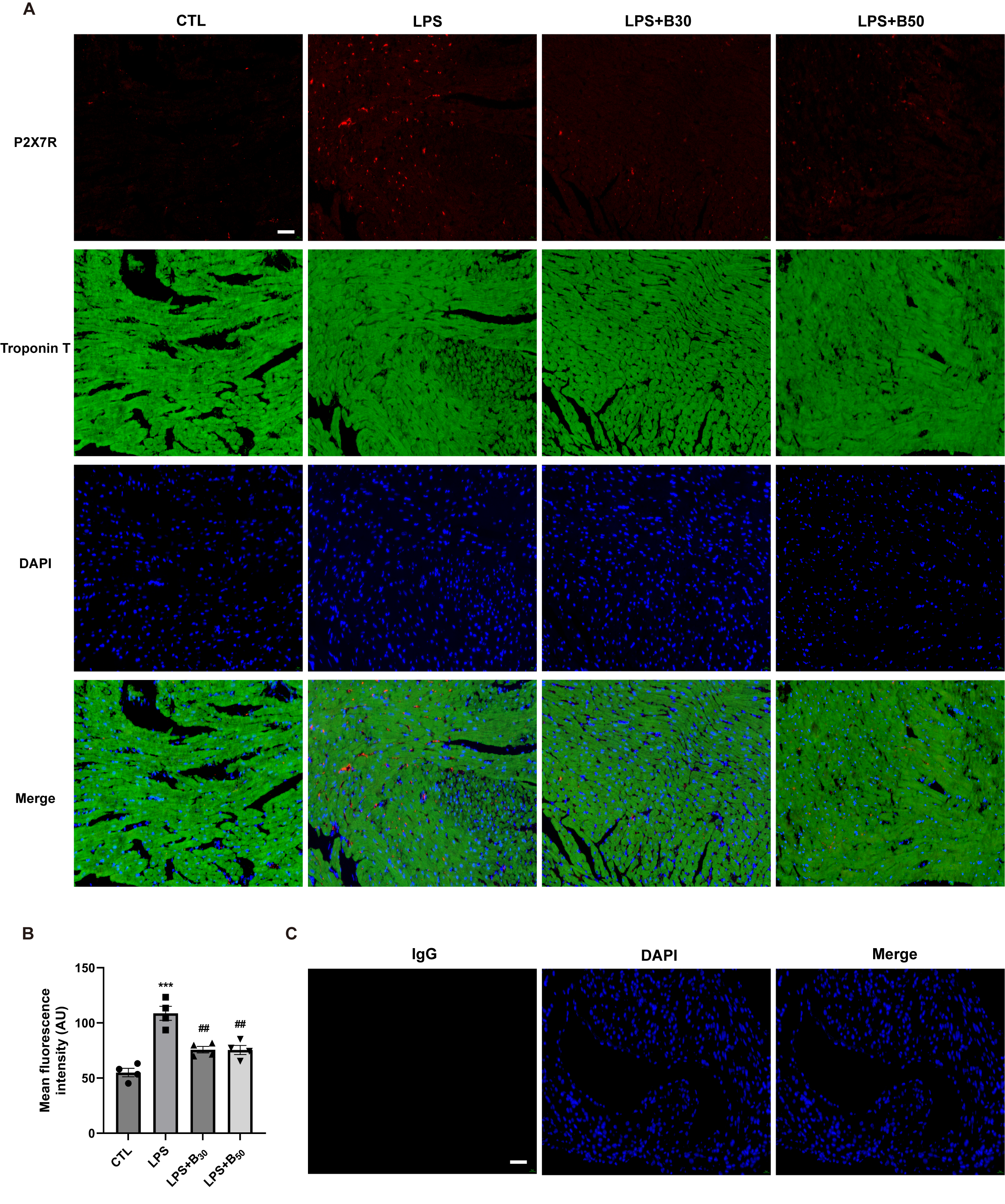

Supplement: euae022_Supplementary_Data [file euae022_supplementary_data.zip › Figure. S1.tif]

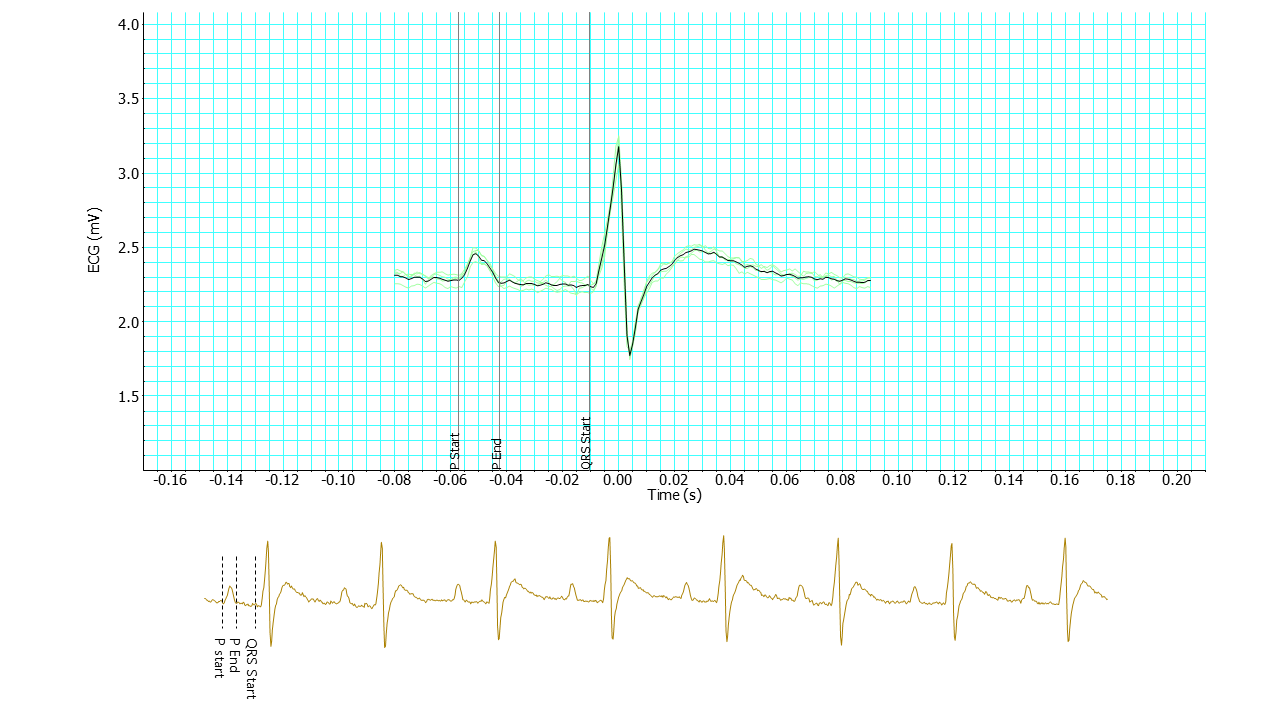

Supplement: euae022_Supplementary_Data [file euae022_supplementary_data.zip › Figure. S2.tif]
